# Intracytoplasmic sperm injection: state of the art in humans

**DOI:** 10.1530/REP-17-0374

**Published:** 2017-12-05

**Authors:** G D Palermo, C L O’Neill, S Chow, S Cheung, A Parrella, N Pereira, Z Rosenwaks

**Affiliations:** The Ronald O. Perelman and Claudia Cohen Center for Reproductive Medicine Weill Cornell Medicine, New York, New York, USA

## Abstract

Among infertile couples, 25% involve both male and female factors, while male factor alone accounts for another 25% due to oligo-, astheno-, teratozoospermia, a combination of the three, or even a complete absence of sperm cells in the ejaculate and can lead to a poor prognosis even with the help of assisted reproductive technology (ART). Intracytoplasmic sperm injection (ICSI) has been with us now for a quarter of a century and in spite of the controversy generated since its inception, it remains in the forefront of the techniques utilized in ART. The development of ICSI in 1992 has drastically decreased the impact of male factor, resulting in millions of pregnancies worldwide for couples who, without ICSI, would have had little chance of having their own biological child. This review focuses on the state of the art of ICSI regarding utility of bioassays that evaluate male factor infertility beyond the standard semen analysis and describes the current application and advances in regard to ICSI, particularly the genetic and epigenetic characteristics of spermatozoa and their impact on reproductive outcome.

## Background

Infertility is defined as the failure to conceive after one year of unprotected intercourse and affects approximately 15% of couples of reproductive age worldwide (Palermo *et al*. 2014*a*). Therefore, the use of ART to treat couples unable to conceive has increased steadily, representing 1.5% of all infants born in the United States (Sunderam *et al*. 2015). Among all indications, male factor infertility is responsible for approximately 50% of couples who are unable to conceive.

Many procedures were developed in the 1980s to address fertilization failure due to dysfunctions of the male gamete, notably zona drilling (Gordon & Talansky 1986), zona softening (Gordon *et al*. 1988, Kiessling *et al*. 1988) or partial zona dissection (Cohen *et al*. 1988) with the most efficient process being subzonal injection of a single spermatozoon into the perivitelline space (Laws-King *et al*. 1987, Palermo & Van Steirteghem 1991, Palermo *et al*. 1992*a*). Indeed, it was during the performance of subzonal injection of an oocyte that the oolemma was accidentally breached and the spermatozoon was delivered into the ooplasm, subsequently establishing the development of intracytoplasmic sperm injection (ICSI) as it is performed still today in humans (Palermo *et al*. 1996*a*).

To date, ICSI (Palermo *et al*. 2014*d*) has been responsible for over two million babies worldwide (ESHRE 2012, Sullivan *et al*. 2013) and has supplanted prior assisted fertilization techniques due to its ability to successfully bypass zona pellucida irregularities and circumvent the presence of antisperm antibodies, sperm acrosome dysfunction and sperm kinetic defects (Palermo *et al*. 1996*c*).

As previously mentioned, ICSI involves the injection of a single sperm cell directly into the ooplasm. The treatment capabilities of ICSI range from the utilization of spermatozoa with poor progressive motility to those gametes microsurgically collected from the epididymis and the testis of azoospermic patients (Palermo *et al*. 1995, 1999, Wallach *et al*. 1996). Beyond male factor, an additional application for ICSI is cases with low oocyte yields. Indeed, ICSI has been used in European countries, such as Germany and Italy, to comply with restrictive laws that limit the number of eggs to be inseminated (Ludwig & Diedrich 1999, Benagiano & Gianaroli 2004). ICSI is also very useful for fertilization of oocytes that were previously cryopreserved (Porcu *et al*. 1997) as cryostress can lead to a premature exocytosis of cortical granules and zona hardening, hindering spermatozoa from penetrating naturally (Johnson 1989, Schalkoff *et al*. 1989, Vincent *et al*. 1990, Van Blerkom & Davis 1994). ICSI is the preferred insemination method to avoid polyspermy, fertilize a high number of oocytes and generate a maximal cohort of embryos. Additionally, the selection of a single spermatozoon significantly reduces the chance of transmission of HIV, HBV and HCV, among others. Indeed, the eventual presence of viruses in semen or accompanying cells may be reduced by the removal of seminal fluid by density gradient preparations and by retrieval of sperm cells directly from a viscous medium just prior injection (Vitorino *et al*. 2011), electing ICSI as the preferential method of insemination for patients at risk for HIV (Peña *et al*. 2002, Sauer & Chang 2002, Mencaglia *et al*. 2005).

ICSI is also unaffected by the immaturity of the male gamete, such as spermatozoa retrieved directly from the epididymis or testis, which are often characterized by an incomplete flagellum and an underdeveloped cell membrane (Palermo *et al*. 1996*c*, 1999). Successful pregnancies from the use of these spermatozoa has pressed the boundaries of the application of ICSI to the most extreme aspect of male infertility, often encountered in cryptozoospermia, virtual azoospermia or of men with absolute azoospermia where surgical retrieval is required (Ron-El *et al*. 1997).

In regard to the popularity of ICSI, a cross-sectional survey of ART procedures performed in 60 countries during 2010 by the International Committee for Monitoring Assisted Reproductive Technologies (ICMART), reported that 63.0% (455,845 of 723,855) of all cycles utilized ICSI (Dyer *et al*. 2016) ranging from a prevalence of 58.4% in Asia to a virtual totality of 98.4% in the Middle East (Dyer *et al*. 2016). Another recent publication, which analyzed ART trends in the United States between 1996 and 2012, reported an increase in the use of ICSI from 36.4% in 1996 to 76.2% in 2012 (Boulet *et al*. 2015). Indeed at our center, there has also been a progressive increase in ICSI utilization starting at 32.2% in 1993 rising to 48.8% in 1995, 73.6% by 2002 and 79.29% in 2016 (Palermo *et al*. 2015*b*, Dyer *et al*. 2016).

The high utilization of ICSI at our center is partly related to the tertiary nature of the clinic and the fact that we are highly integrated with the services of male reproductive urology and surgery. This being the reason why our patients are referred from other clinics, often with a history of several failed ART cycles in terms of fertilization and/or pregnancy. As a note of caution, this should not encourage the indiscriminate utilization of ICSI for cases of non-male factor infertility and should mainly focus on cases with male reproductive dysfunction.

The ability of ICSI to achieve fertilization independently of any observable characteristics of the spermatozoon, although puzzling initially, has guided research into the processes involved in successful fertilization, particularly in cases where dysfunctional spermatozoa have been delivered into the ooplasm. The disparity between the success of ICSI and classic semen parameter thresholds has induced the development of new bioassays aimed at qualifying the male gamete from a genetic and epigenetic point of view. In this review, we describe such pertinent bioassays that investigate the effects on clinical outcome in relation to chromosomal aneuploidy, chromatinic integrity, perinuclear PLCZ responsible for triggering oocyte activation and inducing the initial step of fertilization, the role of the centrosome as a scaffold for the segregation of the first embryonic cleavage, and finally the recently discovered presence of small RNA, that appear imperative post-fertilization to guide embryo development prior to the activation of the embryonic genome. We will also describe the clinical achievements of ICSI throughout the last quarter of a century in regard to the use of spermatozoa of various sources, quality and status considering safety implications on the offspring generated.

## Male gamete bioassays

The semen analysis is the first test that reproductive physicians consult to gain initial information on the male partner’s fertility. Semen analysis is carried out according to the WHO guidelines (WHO 2010) and while the assay measures individual parameters such as semen volume, concentration, motility and morphology of the spermatozoa present, frequent variability among ejaculates in individuals is a recurrent issue (Schwartz *et al*. 1979, Mallidis *et al*. 1991). Moreover, the assessment of semen parameters are subjective and so may appear inconsistent across laboratories (Neuwinger *et al*. 1990, Cooper *et al*. 1992, Matson 1995). It should be noted that the range of normal values published by the WHO are not evidence based and therefore are difficult to interpret in relation to their diagnostic value, resulting in a blanket diagnosis of unexplained infertility that nonetheless can be identified by use of assays with a higher sensitivity (such as the acrosome reaction, antisperm antibody and PLCZ tests). The converse is also true when gametes with suboptimal semen parameters display normal function (Irvine *et al*. 1986, Glazener *et al*. 1987). Due to this disparity between the subjectivity of the semen analysis and WHO standards, it would be advisable for each individual laboratory to identify their own ‘normal’ semen profile.

### High magnification sperm morphology

Traditionally the morphological assessment of spermatozoa has been considered a valuable element (MacLeod & Wang 1979) to predict the fertility potential of infertile men undergoing ART (Kruger *et al*. 1986). However, the introduction of ICSI (Palermo *et al*. 1992*b*) has diminished the relevance of semen parameters in this procedure due to their inability to predict fertilization and pregnancy outcomes in male factor cases (Nagy *et al*. 1995).

Although there is no apparent correlation with clinical outcome, ICSI has shifted the focus from evaluating blanket semen parameters to the observation of each individual male gamete, with the aim of identifying a spermatozoon with normal morphology implying inherent competence in achieving fertilization and supporting embryo development. Selection of individual spermatozoa under high magnification, defined as intracytoplasmic morphologically selected sperm injection (IMSI), is a method used to select spermatozoa that have the choicest morphology in couples with the most severe male factor. IMSI has been proposed in patients with recurrent implantation failure or spontaneous abortions (Lo Monte *et al*. 2013) attributing these pregnancy failures to a sperm defect. However, recent studies have challenged this view, arguing that IMSI does not improve outcomes for couples undergoing a repeated ART attempt (Oliveira *et al*. 2011, Gatimel *et al*. 2016).

In human spermatozoa, it is known that irregular vacuolization of the head is almost ubiquitous (Watanabe *et al*. 2011) and seems to be a paraphysiologic finding (Bedford *et al*. 1973, Tanaka *et al*. 2012), not a sign of incompetency. Irregularities of the sperm head surface occur during spermiogenesis and may have a disparate prevalence in male gametes retrieved from different sites of the male genital tract, and in addition, the appearance and size of the vacuoles may be related to the different stages of capacitation (Kacem *et al*. 2010). Indeed, previous studies have demonstrated that abnormal semen profiles are associated with a modest increase in the frequency of sperm chromosomal abnormalities and that spermatozoa with aberrations in their shape and head contours may be carriers of chromatinic defects (Lewis-Jones *et al*. 2003, Sun *et al*. 2006). However, vacuoles identified on the sperm head are not necessarily related to DNA fragmentation or aneuploidy and therefore, their influence on embryo development remains unclear (Watanabe *et al*. 2011). Moreover, the issue of safety remains paramount to address, indeed infants born from this procedure tend to have a higher occurrence of low birth weight (<2500 g) (Junca *et al*. 2010).

Although different high-power magnification techniques corroborated by video-generated magnification have been suggested to deselect dysmorphic spermatozoa, these methods are inherently limited by the clarity of the image, the time required for image analysis and the risk of variable spermatozoa head-positioning during imaging due to the asymmetry of the head contour (Palermo *et al*. 2011).

While the current concept of IMSI is still being debated and further augmented, the interest in the meticulous assessment of the spermatozoon, its contour and all its facets remains an intriguing quest. To address these observational issues, a study was performed at our center aimed at eliminating the aforementioned limitations on high magnification sperm morphology, in which an image-tracking software was used to capture serial photographs of spermatozoa from recorded videos. The images were automatically extracted from each digital frame using enhanced correlation coefficient maximization; the general shape of the spermatozoa was then extracted via space-carving. The reconstructed image was rotated to permit viewing from any vantage and the final image was rendered via interpolation. This method yielded images that enable noninvasive, 3-D, real-time, *in vitro* assessment of sperm surface morphology that is easily automated and required little equipment, presumably available in most embryology laboratories. From this study, we observed that although a spermatozoon may appear to be morphologically acceptable by IMSI standards, when rotated, the head of the cell revealed a vacuole, suggesting that even spermatozoa selected via high magnification for injection may still possess the same characteristics as the spermatozoa deselected in the IMSI procedure (Levine *et al*. 2015).

While the selection of the sperm is undoubtedly the proper approach, it is clear that physical characteristics do not provide any specific information on the health of the genome and epigenome of the male gamete. Therefore, further screening needs to be employed to truly quantify the competence of the spermatozoa.

### Sperm aneuploidy assessment

High fetal wastage in humans is commonly attributed to aneuploidy. Most aneuploid pregnancies do not survive, with the majority of losses occurring during the first few weeks of uterine life. In general, autosomal trisomies constitute the large majority of aneuploid embryos, with 16, 18 and 21 having a maternal origin and sex chromosomal aneuploidies (45X, 47XXY, 47XYY) often originating from the paternal origin (Hassold & Hunt 2001, Neri *et al*. 2014*a*). While meiotic errors that lead to fetal aneuploidy can originate from either the male or female gamete, the occurrence is lower in spermatozoa (9%) when compared to oocytes (20%) (Hassold & Hunt 2001, Neri *et al*. 2014*a*). Regardless, assessment of male gamete ploidy is an important aspect of pre-fertilization genetic diagnosis.

The assessment of sperm chromosome abnormalities has increased over the years with the popularity of ICSI, especially after it was recognized that infertile men possess an increased frequency of sperm aneuploidy despite having a normal peripheral karyotype (Martin 2006). It was previously demonstrated that men with suboptimal semen parameters have an increased frequency of sperm chromosomal abnormalities (Colombero *et al*. 1999*a*). Furthermore, chromosomal evaluation of the aging male gamete has become of particular interest and is crucial in determining the extent of meiotic errors, which can affect the conceptus’ health. In studies performed on ejaculated spermatozoa, the occurrence of chromosome 18 disomy has been found to be significantly higher in men over 50 years of age (Griffin *et al*. 1996). There was also a linear increase in chromosome 9 disomy with respect to age (Bosch *et al*. 2003). Similar results were reported for chromosome 21 (McIntosh *et al*. 1995), where a higher frequency of disomy 21 was identified in men more than 60 years of age (Rousseaux *et al*. 1998). Chromosome 1 disomy was reported to increase with advancing paternal age, while the assessment of gonosomal disomy observed an increase in disomy YY (Martin *et al*. 1995). There was also a significant relationship established between increasing paternal age and XY disomy (Asada *et al*. 2000).

These findings, however, were not always confirmed by other studies. For instance, in a separate study, there was no correlation found between paternal age and chromosome 12, XX or XY disomy (Martin *et al*. 1995). Even more contradictory, chromosome 13 and 18 disomy have both been found to increase in younger men instead (Steiner *et al*. 2015). Furthermore, another study found that although aneuploidy of the gonosomes is more common than that of autosomes, neither occur more frequently with increasing paternal age (Donate *et al*. 2016).

**Figure 1:**
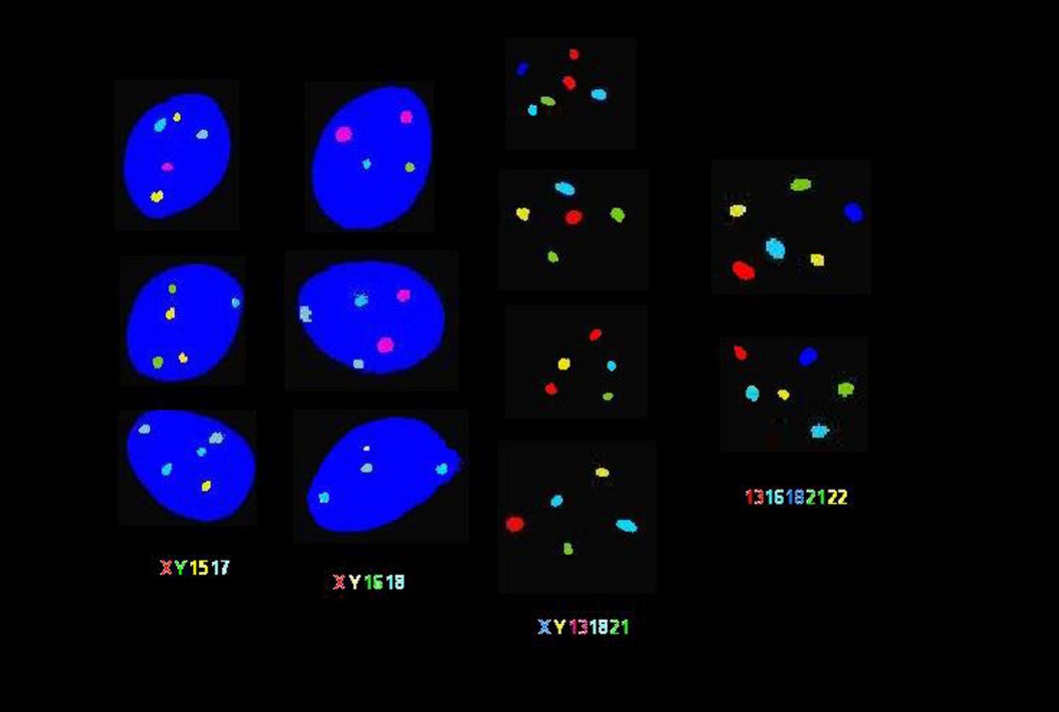
Fluorescent *in-situ* hybridization (FISH) analysis of ejaculated human spermatozoa. FISH analysis was carried out using 4 different probe sets. In the 2 columns on the left, sperm chromatin stained with 4′,6-diamino-2-phenylindole (DAPI) appears in blue. As indicated from left to right: spermatozoa were assessed by probe sets for chromosomes X/Y/15/17, X/Y/16/18, X/Y/13/18/21 and 13/16/18/21/22 in various colors. As depicted in each cell, disomy is indicated by the appearance of multiple fluorescent signals in the same color. Spermatozoa exhibiting various occurrences of gonosomal and autosomal disomy are shown.

Although fluorescent *in situ* hybridization (FISH) enables the detection of chromosomal abnormalities, the indications for FISH on sperm are currently not fully established (Funaro & Paduch 2014). At our center, patients with a history of recurrent ART failure or recurrent pregnancy loss are assessed for sperm aneuploidy. This assessment is particularly stressed for azoospermic patients who are undergoing epididymal and testicular retrieval. However, with FISH, only a limited number of chromosomes can be analyzed and only specific regions of interest labeled by the fluorescent probe may be visualized (Fig. 1) (Neri *et al*. 2014*a*). Twenty-four chromosome FISH on spermatozoa may be a more advantageous method to determine the overall aneuploidy in a particular specimen (Hu *et al*. 2011). Furthermore, a more accurate aneuploidy assessment can be achieved by determining specific copy number variants of sequenced DNA in individual spermatozoa.

### Sperm chromatin assessment

During late spermiogenesis in the testis, male gametes undergo a complex and sensitive process of chromatin condensation, in which DNA strands are severed in order to allow tight supercoiling around protamines, owing to the action of testis-specific serine kinase 6 (TSSK6) (Palermo *et al*. 2014*c*). The chromatin packing of sperm cells is markedly different from that in somatic cells, primarily differentiated by the substitution of protamines for DNA compaction rather than histones (Ward 2010). Said protamines are introduced through an exchange with histones, regulated by the H1 histone family, member N, testis specific (H1FNT) to allow for a tighter compaction of the chromatin. The spermatozoa’s DNA is densely wrapped for its protection, rendering the spermatozoa transcriptionally inactive and resilient to damage during transport through the male genital tract and subsequently, the female genital tract. In addition to providing protection while in transit through the uterine environment, nuclear compaction results in a hydrodynamicity of the head, allowing for better sperm mobility and penetration through the zona pellucida of the ovum (Dadoune 2003).

Although the majority of spermatozoon DNA is tightly bound around protamines, between 2% and 15% of the chromatin is bound in histone linker sections (Dadoune 2003, Hammoud *et al*. 2009*b*) that can be found throughout the genome, specifically at gene promoter regions (Wykes & Krawetz 2003). The family of genes involved in embryo development has been observed to preferentially persist on residual histone regions in human spermatozoa (Hammoud *et al*. 2009*b*). This finding demonstrates that histones, rather than being distributed haphazardly in the sperm genome, are linked to specific genes and compose conserved linker regions with high nuclease sensitivity between each protamine-bound toroid (Sotolongo *et al*. 2003). It is these histone linker regions that are actually assessed by the majority of chromatin status tests (Noblanc *et al*. 2013).

Understanding this unique process of chromatin packing is essential in the development of tests for male infertility and the assessment of sperm chromatin characteristics, which may have distinct consequences on ART outcomes (Evenson & Wixon 2006, Zini & Sigman 2009). Several studies have suggested that fertile men with normal semen analyses generally have lower levels of DNA breakage than infertile men, in particular those with compromised semen parameters. However, up to 8% of infertile men with compromised DNA integrity may present with normal parameters for concentration, motility and/or morphology (Zini *et al*. 2001).

Thus, the origin of DNA damage in the male gamete seems to be caused by a multitude of inherent and external factors. Protamine deficiencies and DNA packaging defects (Hammoud *et al*. 2009*a*) comprise just a small number of the inherent factors that indicate the potential for DNA damage of this cell.

Separately from innate defects that can affect chromatin integrity, advanced age in men has been found to be associated with a higher incidence of sperm DNA damage (Plastira *et al*. 2007, Sakkas & Alvarez 2010, Zanko *et al*. 2014). In addition, environmental factors such as cigarette smoking, (Künzle *et al*. 2003) genital tract inflammation, varicoceles (Saleh *et al*. 2003) and hormone deficiencies (Xing *et al*. 2003) are correlated with increased DNA damage, as described in both human and animal studies (Mathur & D’Cruz 2011, Lewis *et al*. 2013).

It is postulated that spermatozoon DNA integrity is closely associated with sperm quality, male fertility potential and pregnancy outcomes (Brewer *et al*. 2002). Specifically, an abnormal DNA fragmentation index (DFI) is thought to have an inverse relationship with male fertility success (Zini *et al*. 2001) and if pregnancy does occur, these conceptuses are thought to be at an increased risk of miscarriage (Kumar *et al*. 2012).

**Figure 2:**
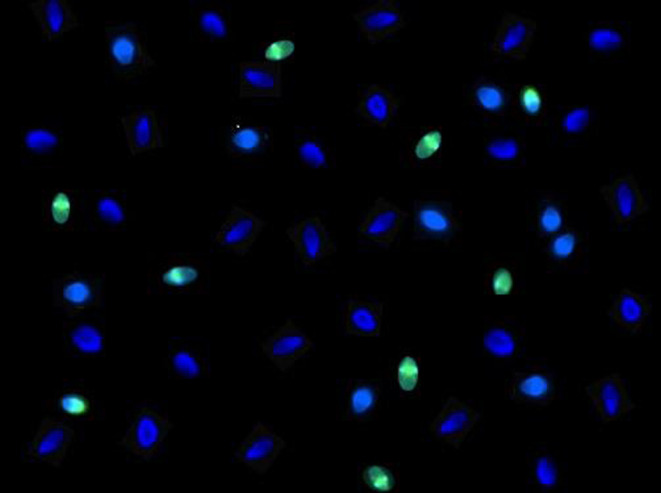
Terminal deoxynucleotidyl transferase dUTP nick-end labeling (TUNEL) immunofluorescent staining of human spermatozoa for detection of DNA fragmentation. Sperm cells which fluoresce green indicate the presence of DNA fragmentation that occurs during the late stages of apoptosis, detected by the action of the TdT enzyme. Spermatozoa are considered TUNEL positive if approximately 40% or more of the head is fluorescent. Spermatozoa without compromised chromatin integrity are shown in blue using DAPI counterstain. Five hundred sperm cells per sample are assessed using fluorescent microscopy in order to determine the sperm chromatin fragmentation, with a threshold of ≤15% TUNEL positivity considered normal.

The most popular techniques for evaluating sperm DNA integrity include the sperm chromatin structure assay (SCSA), terminal deoxynucleotidyl transferase (TdT) dUTP Nick-End Labeling assay (TUNEL) (Fig. 2), sperm chromatin dispersion test (SCD) and the comet assay (Funaro & Paduch 2014). Although there has been little standardization within chromatin integrity assays and a high variability among index thresholds per assay and per testing laboratory, SCSA, TUNEL and SCD have been found to have comparable predictive values for DNA fragmentation (Chohan *et al*. 2006). Although these tests provide invaluable information regarding the chromatin integrity of an individual spermatozoon, it is important to note that these assays are consumptive; in other words, once the sample is used for any of the aforementioned assays, it cannot be used for the treatment of the couple (Funaro & Paduch 2014). Thus, in current clinical practice, it is difficult to isolate spermatozoa with a high level of chromatin integrity for use at the time of insemination (Funaro & Paduch 2014) as characteristics such as an abnormal motility or morphology of the spermatozoon in question, does not directly signify impaired chromatin integrity (Chen *et al*. 2011, Palermo *et al*. 2015*a*).

With that being said, DNA fragmentation as assessed by the most popular assays, does correlate with semen parameters of motility and morphology *en masse* (Younglai *et al*. 2001, Tang *et al*. 2010); however, this correlation is not predictive of pregnancy outcomes in relation to ICSI insemination as corroborated by Evenson and Wixon (Evenson & Wixon 2006) and by Zini (Zini 2011). ICSI insemination is *sui generis* for sperm transport, eliminating the effects of sibling spermatozoa. Thus, the selection of the best-looking, progressively and regularly motile spermatozoon may account for the lack of correlation between DFI values and pregnancy rates, as it has been found in several studies that utilization of the motile portion of ejaculated spermatozoa with *in vitro* insemination methods curtails DNA-damaged spermatozoa from generating a conceptus (Zini *et al*. 2008, Aitken *et al*. 2009, Simon & Lewis 2011, Palermo *et al*. 2014*c*).

With that being said, a recent meta-analysis of 16 IVF studies and 24 ICSI studies using TUNEL, SCSA, COMET or SCD did suggest that there is an effect of DNA fragmentation on IVF and ICSI outcomes (Simon *et al*. 2017). However, the majority of the studies included relating DNA fragmentation to ICSI did not control for confounding female factors. These include advanced maternal age that may result in oocytes with a compromised ability to repair DNA damage of spermatozoa (Sakkas & Alvarez 2010, Meseguer *et al*. 2011). Additionally, it was noted in another recent meta-analysis assessing the four most popular chromatin integrity assays mentioned above that, while the TUNEL and COMET tests were of fair predictive power in regard to clinical pregnancies with IVF and ICSI once compared to SCSA and SCD, the specificity of their predictive power was low suggesting that even levels of DNA fragmentation below threshold did not guarantee a successful pregnancy (Cissen *et al*. 2016). Considering these factors, while a valuable understanding of the effects of DNA fragmentation is beginning to form, widely accepted standards for each assay need to be assigned before we can more accurately evaluate the impact of DNA fragmentation on ART procedures.

### PLCz

Despite the widespread success of ICSI treatments for male factor infertility, cases of complete fertilization failure continue to persist. Their occurrence is emotionally devastating for couples, with significant expenditure of logistic and financial resources to no avail. Therefore, understanding the etiology of fertilization failure is of critical importance to counsel patients and devise a successful treatment protocol.

At our center, ICSI with ejaculated spermatozoa yields a fertilization rate well over 70%, irrespective of sperm characteristics. It seems the only requirement for fertilization is that the spermatozoon displays some level of motility and certainly maintenance of viability (Palermo *et al*. 2009). With that being said, couples undergoing ICSI can still experience a total lack of fertilization, with a frequency of occurrence just below 3% (Palermo *et al*. 2015*a*). Complete fertilization failure can be due to various etiologies such as unsuccessful immobilization and breaching of the spermatozoal membrane, a lack of decondensation of the sperm nucleus or a failure of the oocyte to undergo activation (Sousa & Tesarik 1994, Flaherty *et al*. 1995, Palermo *et al*. 1996*c*, Yanagida 2004), although most often, the rationale can be attributed to an oosplasmic dysmaturity (Pereira *et al*. 2016*b*). Nonetheless, in a very small proportion of cases, recurrent failed fertilization is a result of the inability of the male gamete to activate an oocyte due to a lack of an activating cytosolic factor (Swann *et al*. 2004, Kashir *et al*. 2010, Neri *et al*. 2014*b*).

Our team has been among many that attempted to identify and correct for a sperm cytosolic oocyte-activating factor (Palermo *et al*. 1997, Wolny *et al*. 1999), attributing its absence to the reason why certain infertile men fail to fertilize their partner’s oocytes (Neri 2010, Neri *et al*. 2010).

Over a period of 23 years at our center, ICSI was performed in 19,757 couples, of which 2.6% experienced a complete failure of fertilization. In a small portion of these couples, the absence of an oocyte-activating factor in the spermatozoa was reported by PLCZ (phospholipase c-zeta) assessment (Neri *et al*. 2010), distinguishing cases with an oocyte-activating factor deficiency vs an ooplasmic dysmaturity (Neri *et al*. 2014*a*,*b*, Pereira *et al*. 2016*b*).

Oocyte activation is induced by a sizeable influx of calcium into the cell, stimulating the different pathways required for proper fertilization and prevention of polyspermy (Tosti & Ménézo 2016). It has been recently demonstrated in animal studies that PLCZ found in the perinuclear theca of the spermatozoa is the factor responsible for oocyte activation in mammals. A study in mice used the CRISPR-Cas9 system in order to create *Zfy1/2* double-knockout mice, significantly decreasing PLCZ expression and producing mice with spermatozoa that failed to fertilize oocytes after ICSI (Nakasuji *et al*. 2017). In a second study also using CRISPR-Cas9 gene editing technology, investigators were able to create *Plcz1*-null mice and tested their fertilizing capability using IVF and ICSI (Hachem *et al*. 2017). When compared to the wild type, Plcz-null mice gametes had significantly higher events of polyspermy in IVF and significantly lower instances of normal fertilization and embryo development after ICSI (Hachem *et al*. 2017). Therefore, in the event of recurrent fertilization failure, it would be useful to screen male patients for the presence of PLCz in spermatozoa. Couples that have experienced fertilization failure as a result of low expression of PLCz in the male partner’s gametes then have the option to undergo assisted oocyte activation (AOA) (Neri *et al*. 2010). Although the prevalence of such cases using AOA are low and often result in little success, the use of calcium ionophore treatment in these patients can improve fertilization results and even progress safely to pregnancy (Chi *et al*. 2004, Sugaya 2010, Neri *et al*. 2014*b*).

### Centrosome

During the process of fertilization, fusion of the two parental haploid chromosomal complements allows for the formation of the diploid genome of the conceptus. In humans, the mature oocyte contains the supporting elements capable of sustaining the development of the embryo, while a primary contribution of the spermatozoon is to provide the centrosome, the scaffold that generates the first mitotic spindle and ordains a correct chromosomal segregation for this new entity. In one of the early studies at our center, we were able to define the contribution of the male gamete in supplying the centrosome to the oocyte for proper embryonic cleavage (Palermo *et al*. 1994). The centrosome consists of a pair of centrioles carried at an angle from each other, the proximal centriole, comprising nine triplets of microtubules located at the base of the sperm head, and the distal centriole, representing the main framework for the development of the flagellum (Sathananthan *et al*. 1991, 1996, Neri *et al*. 2010). The use of immunological techniques allows for the identification of proteins that are intimate components of this microtubule organizing center (Kimble & Kuriyama 1992, Palermo *et al*. 1997, Colombero *et al*. 1999*b*, Neri *et al*. 2011). Indeed, a centrosomal defect results in embryonic aneuploidy or mosaicism resulting in the inability of the conceptus to undergo the first embryonic cleavage effectively (Moomjy *et al*. 1999). In cases where syngamy fails to occur after ICSI or a chaotic distribution of the chromosomes within the conceptus is identified, the spermatozoon centriole can be labeled with anti-centrin antibodies to assess for its presence and estimate its integrity by measuring the angle that the proximal centriole creates with the distal centriole of the flagellum (Neri *et al*. 2011). Indeed, the angle appears to be unique to the centriolar geometry within the human spermatozoon. Several studies have identified cases of the centrosome being severely altered or absent in infertile men, plagued by abnormal midpieces and stump tails originating in the testis and often resulting in complete immotility of the spermatozoa in the ejaculate (Baccetti *et al*. 1989, Barthelemy *et al*. 1990, Stalf *et al*. 1995, Toyama *et al*. 2000, Gambera *et al*. 2009, Neri *et al*. 2011). Although there is a high rate of centrosomal abnormality in these ejaculate specimens, the use of ICSI with these immotile spermatozoa can result in normal fertilization and pregnancies (Stalf *et al*. 1995, Barros *et al*. 1997). This confirms the importance of the male gamete to contribute in generating an euploid conceptus, and therefore, the availability of an assay capable of estimating centrosomal integrity and function is undeniable.

Recent studies have identified that variants in maternal *PLK4*, normally responsible for mediating centriole duplication and embryo cleavage, result in aneuploidy within the embryo (McCoy *et al*. 2015). Furthermore in a recent study, assessment of a cohort of infertile women evidenced that a common variant rs2305957 of the *PLK4* gene yielded a significantly lower blastocyst rate in comparison to a control; this variant was particularly pronounced in a subset of patients with early recurrent miscarriage (Zhang *et al*. 2017).

The understanding of the role of the centrosome in the human male gamete, made possible by a shift in focus to the competence of a single spermatozoon induced by ICSI, has heightened the study of the spermatozoon as a contributor not only of the paternal genome, but as a vector of an important organelle that ordains the chromosomal segregation during the first mitotic division.

### Sperm small RNA

The recent discovery of RNA present in human spermatozoa has raised several interesting questions regarding its role in male fertility (Krawetz *et al*. 2011, Jodar *et al*. 2013). Analyses of spermatozoal RNA transcripts have shown to contain remnants of prior events in spermatogenesis as well as highlight potential genes that may be critical for fertilization and embryo development (Krawetz *et al*. 2011, Jodar *et al*. 2013). In addition to mRNA, human spermatozoa carry small non-coding RNAs (sncRNAs), in which the distribution in ejaculated specimens is as follows: 65% repeat-associated small RNAs, 17% Piwi-interacting piRNAs, 11% quiescent RNAs and 7% microRNAs (Krawetz *et al*. 2011). Evidence of sncRNAs suggests a role of spermatozoal transcripts in post-fertilization development and further designates them as an emerging biomarker of male infertility (Krawetz *et al*. 2011).

The first specific mRNA identified in human spermatozoa was *C-MYC* mRNA (Kumar *et al*. 1993), thereafter several studies utilizing RT-PCR (reverse transcriptase PCR) or ISH (*in situ* hybridization) identified specific transcripts encoding protamines, progesterone and estrogen receptors, CYCLIN B1, STAT4, DAZL, SRY and PLCZ (Dadoune 2009). Additionally, ISH was used in studies to pinpoint the localization of such RNA at the periphery of the nucleus beneath the nuclear envelope, revealing an interior component of the nuclear matrix previously unexplored (Hamatani 2012). The presence of such mRNAs consistently persisting in ejaculated spermatozoa suggests a purposeful conservation of these transcripts post-spermiogenesis (Ostermeier *et al*. 2002, Miller & Ostermeier 2006). Indeed, several conserved transcripts in spermatozoa between mammalian species have been recently identified, suggesting an important role of these RNA in the spermatozoon’s contribution to early embryonic development (Schuster *et al*. 2016). An example of such transcripts contributing to the oocyte is the identification of RNA encoding the previously discussed PLCZ, which upon isolation and injection into a mouse oocyte, triggered calcium oscillations and activation of the oocyte (Sone *et al*. 2005).

An important role attributed to spermatozoal RNA is the epigenetic reprogramming of the sperm chromatin (Miller *et al*. 2005), acting as a stabilizer for the interaction between the nuclear envelope and the small regions of histone-bound DNA, as well as mediating the selective escape of these histone-bound sequences from tight packing around protamines, therefore influencing the balance between protamine- and histone-packaged DNA (Hammond *et al*. 2009*a*,*b*). Epigenetic studies are foreseen to play an increasingly important role in the etiology of human infertility (Carrell 2012) as epigenetic regulation is becoming apparently more useful in quantifying the impact of environmental factors on the male gamete (Furrow *et al*. 2011).

Gene expression is regulated post-transcriptionally via small non-coding RNA entities as well as miRNA, responsible for fine-tuning cell differentiation through translational regulation during spermatogenesis (McIver *et al*. 2012). Further, antisense RNA has been identified in the human male gamete and includes small antisense RNAs, PIWI-interacting RNAs (piRNAs), MIWI and germline-specific argonaute proteins involved in RNA silencing (Ostermeier *et al*. 2005*a*,*b*, Girard *et al*. 2006, Grivna *et al*. 2006, Kim 2006). This small antisense RNA carried by the sperm cells suggest that paternal ribonucleic acid may help regulate embryonic gene expression (Biermann & Steger 2007, Boerke *et al*. 2007). Further, non-coding RNA have been found to be involved in the X-inactivation occurring in the spermatocyte during spermatogenesis, thus representing a large complex in ejaculated human spermatozoa that may also orchestrate gene expression upon fertilization (Royo *et al*. 2015).

There have already been preliminary efforts into classifying fertile and infertile men via the analysis of their respective spermatozoal transcriptomes. Microarray analysis comparing oligozoospermic infertile men to normozoospermic fertile men revealed a remarkable down-regulation of genes relating to germ cell anti-apoptotic mechanisms (*PRN2*, *SP2-1*, *STATA-4*, *MRA-1*, *CREM*), a reduced expression of DNA repair (*NIPBL*), oxidative stress regulation (*PARK-7*), histone modification (*DDX3X*), spermatogenesis and sperm motility (Montjean *et al*. 2012). RNA profiling by microarray was also carried out in infertile men with normal semen parameters reporting a several-fold reduction in the expression of 136 genes (Garrido *et al*. 2009). In another study assessing infertile men with teratozoospermia in comparison to a cohort of men that had previously fathered a child, teratozoospermic men were found to lack RNA involved in the ubiquitin-proteasome pathway as well as other transcripts involved in acrosomal development and oocyte activation (Platts *et al*. 2007).

Furthermore, transcriptome analysis is already being applied to studying clinical outcomes in infertile men. A study of a small cohort of patients undergoing their first cycle of intrauterine insemination (IUI) treatment screened for over 19,000 transcripts, reporting that the cohort supporting a pregnancy had 756 overexpressed genes while only 194 of these common transcripts were overrepresented in couples that did not achieve a pregnancy (Garcia-Herrero *et al*. 2010). Additionally, expression of 741 transcripts was identified as exclusive to the fertile cohort. These findings are also in concordance with a more recent study evidencing that the absence of certain sperm RNA elements (SREs), as assessed in 96 couples with idiopathic infertility, were predictive of clinical outcome, reducing timed intercourse and IUI outcome success rates from 73% to 27% (Jodar *et al*. 2015).

In men with unexplained infertility, supplementary tests are pivotal to gaining insight into the paternal contribution to the zygotic genome. Profiling men via RNA sequencing to supplement the standard semen analysis may aid in the diagnosis and management of couples with recurrent ART failures. Although further research on spermatozoa-borne transcripts is needed, based on findings to date, screening men for an epigenetic imbalance of sncRNA and lncRNA may provide crucial information on the etiology of unexplained infertility and overall reproductive capacity of the infertile male.

## 25 years of ICSI

In this section, we will recount our experience at our center using ejaculated, epididymal and testicular spermatozoa with ICSI over the last quarter of a century; data have been updated from studies by Palermo and coworkers ( Palermo *et al*. 2012, 2014*b*). To summarize our overall clinical data from ICSI, of a total of 35,065 cycles, 15,646 cycles presented with a positive βhCG (44.6%), resulting in losses of 2694 biochemical (17.2%) and 861 blighted ova (5.5%). Among the 11,548 cycles that progressed to clinical pregnancy as defined by the observation of a fetal heartbeat, 1343 resulted in additional losses from miscarriage or therapeutic abortion. These overall cases resulted in a pregnancy and delivery rate of 32.9% per retrieval (11,548/35,065) and 38.1% per embryo replacement (11,548/30,289), resulting in the birth of 12,719 neonates from 9572 deliveries, consisting of 6230 females and 6315 males. The frequency of multiple deliveries including 2711 twins was (28.3%), 212 triplets (2.24%), and 4 quadruplets (0.04%).

### Ejaculated spermatozoa

Between September 1993 and April 2017, a total of 31,723 ICSI cycles using ejaculated spermatozoa were performed. To provide an overview, a total of 262,659 MII oocytes were injected; 75.7% fertilized normally while 2.5% and 3.8% were 1PN and 3PN respectively, with no fertilization observed in 14.4%. The clinical pregnancy rate per oocyte retrieval was 36.4% and per embryo transfer procedure was 42.1%.

The advantages ICSI has provided over other ART techniques for severe male factor patients have been well documented in the literature over the past 25 years. Indeed, a study assessing a cohort of men with asthenoteratozoospermia reported a pregnancy rate of just 8% with IUI, which increased to 29% with ICSI (Mangoli *et al*. 2008).

Additionally, oligozoospermic patients with a count less than 5 million spermatozoa/mL in the neat sample have been found to have a pregnancy rate as low as 4% with intrauterine insemination (Mangoli *et al*. 2008). Our patient population includes many severely oligozoospermic men with a concentration less than 1 × 10^6^/mL spermatozoa, therefore signifying a primary indication for ICSI. In 1969 ejaculates where the initial specimen presented with a count less than 1 million/mL, a high-speed centrifugation was carried out, resulting in an average sperm density of 0.85 ± 2.6 × 10^6^/mL and a motility of 29.8 ± 29%. Subsequent use with ICSI yielded a fertilization rate of 61.1% and a clinical pregnancy rate of 40.0% (Table 1).

**Table 1:** ICSI outcomes in men with severe oligozoospermia (<1 × 10^6^/mL spermatozoa).

**Parameter**	**Value**
Cycles	1969
Mean initial concentration (10^6^ per mL ± s.d.)	0.2 ± 0.2
Mean initial motility (% ± s.d.)	17.8 ± 21.8
Mean morphology (% ± s.d.)	0.7 ± 1
Fertilization (%)	11,036/18,067 (61.1)
Clinical pregnancy (%)	788 (40.0)

### Surgically retrieved spermatozoa

The value of ICSI chiefly lies in the ability to establish a successful pregnancy with surgically retrieved specimen from azoospermic men. Prior to the implementation of ICSI, such situations were resolved with the use of donor spermatozoa. Azoospermia presents in less than 1% of men and in about 10–15% diagnosed with infertility, either as obstructive (OA) or non-obstructive (NOA) azoospermia (Hamada *et al*. 2013). Obstructive azoospermia can be due to a congenital bilateral absence of the vas deferens (CBAVD) often linked to a defect in the CTFR gene associated with cystic fibrosis (Patrizio & Zielenski 1996), trauma, infection, or vasectomy (Hamada *et al*. 2013). Specimen to be utilized for ICSI may be aspirated from the epididymis (MESA) or retrieved percutaneously (PESA) (Kahraman *et al*. 1996, Schlegel *et al*. 1997, Schlegel & Li 1998). In men with non-obstructive azoospermia in which spermatogenesis is scant, a testicular sperm extraction (TESE) or a micro-TESE (mTESE) can yield adequate spermatozoa while maintaining anatomical integrity (Schlegel 2009). Additionally, epididymal and testicular sampling can also be an effective alternative in males with extreme oligoasthenoteratospermia (OAT) (Palermo *et al*. 1992*b*, 2014*a*, Nyboe Andersen *et al*. 2009). Furthermore, men with cryptozoospermia that undergo a surgical retrieval have resulted in improved clinical outcomes compared to their previous cycle with ejaculated specimen (Bendikson *et al*. 2008, Ketabchi 2016).

**Figure 3:**
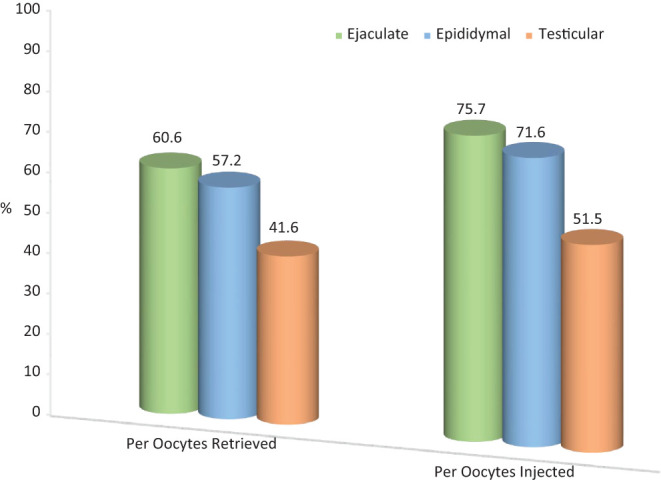
Comparison of fertilization rates according to spermatozoa source. Ejaculated specimens yielded a fertilization rate comparable to the epididymal and both were superior to testicular spermatozoa per oocytes retrieved (*χ*^2^, 2 × 3, 2 *df*; *P* < 0.0001). A similar pattern was observed once the fertilization rate was calculated based on the number of metaphase II oocytes injected (*χ*^2^, 2 × 3, 2 *df*; *P* < 0.0001).

**Figure 4:**
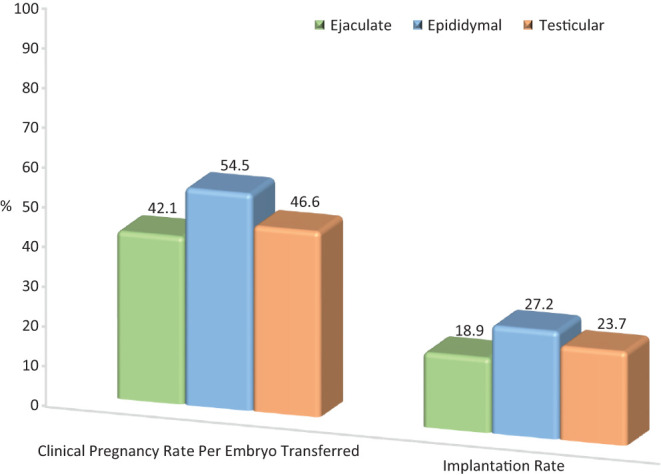
Comparison of pregnancy and implantation rates according to spermatozoa source. Embryos generated from epididymal spermatozoa had the highest pregnancy rate followed by testicular and ejaculated spermatozoa (*χ*^2^, 2 × 3, 2*df*; *P* < 0.0001). Embryo implantation rate was highest with epididymal, followed by testicular and ejaculated specimen (*χ*^2^, 2 × 3, 2 *df*; *P* < 0.0001).

The use of spermatozoa sourced from different areas within the male genital tract has led to an increased scrutiny in regard to fertilizing ability and clinical outcome. Over the past 25 years, our center has carried out 1140 cycles using epididymal spermatozoa and 1713 cycles with testicular spermatozoa. Table 2 summarizes the patient demographic and embryologic data of couples inseminated with different spermatozoa sources, chiefly ejaculated, epididymal, and testicular. Epididymal spermatozoa yielded the highest number of fertilized oocytes per cycle, followed by the ejaculate and testicular spermatozoa (Fig. 3). Although having a lower fertilization rate, testicular spermatozoa demonstrated a significantly higher pregnancy rate compared to the ejaculated specimen (Fig. 4), which has also been confirmed in other studies (Esteves *et al*. 2015, Pabuccu *et al*. 2017).

**Table 2:** Outcomes using ejaculated, epididymal and testicular spermatozoa. Data are presented as mean ± s.d.

**Parameter**	**Ejaculated**	**Epididymal**	**Testicular**
Maternal age (years)	37.7 ± 5^a^	35.3 ± 5^b^	33.8 ± 6^c^
Cycles	31,723	1140	1713
Oocytes retrieved	10.3 ± 6^d^	12.0 ± 7^e^	12.5 ± 7^f^
Oocytes injected	8.3 ± 5^g^	9.6 ± 5^h^	10.1 ± 5^i^
Oocytes fertilized	6.3 ± 4^j^	6.9 ± 5^k^	5.2 ± 4^l^
Clinical pregnancy rate per cycle (%)	11,536 (36.4)^m^	576 (53.2)^n^	687 (40.1)^o^

a vs b vs c: ANOVA, 2 *df*, effect of sperm source on average maternal age, *P* < 0.0001; d vs e, f: *t*-test, 1 *df*, effect of sperm source on number of oocytes retrieved, *P* < 0.0001; g vs h, i: *t*-test, 1 *df*, effect of sperm source on number of oocytes injected, *P* < 0.0001; j vs k vs l: ANOVA, 2 *df*, effect of sperm source on number of oocytes fertilized, *P* < 0.0001; m vs n vs o: *χ*^2^, 3 × 2, 2 *df*, effect of sperm source on clinical pregnancy rate, *P* < 0.0001.

Implantation rates were congruent with the clinical pregnancy rates by sperm source, with epididymal specimens yielding the highest implantation rate, followed by testicular, and finally ejaculated specimen. In our experience, utilization of fresh testicular spermatozoa with ICSI yielded a more consistent zygote development and clinical pregnancy rate in comparison to the cryopreserved counterpart (Table 3), which appears to be discordant with the findings of other studies that did not see a significant difference (Habermann *et al*. 2000, Ohlander *et al*. 2014, Schachter-Safrai *et al*. 2017). Although reaching comparable fertilization rates, thawed epididymal spermatozoa had impaired motility and lower pregnancy outcomes than the fresh counterpart (Table 3). It must be noted that this analysis is purely academic, due to the fact that spermatozoa retrieved from different sources address different clinical indications.

**Table 3:** Spermatozoal parameters and intracytoplasmic sperm injection outcome according to retrieval sites and specimen condition.

**No. of items**	**Spermatozoa**			
	Epididymal		Testicular	
	Fresh	Frozen/thawed	Fresh	Frozen/thawed
Cycles	364	776	1158	555
Density (10^6^ per mL ± s.d.)	37.9 ± 44	21.1 ± 26	0.3 ± 2.6	0.3 ± 1.8
Motility (% ± s.d.)	19.0 ± 17^a^	3.9 ± 9^a^	2.9 ± 7	1.4 ± 5
Morphology (% ± s.d.)	1.7 ± 2	1.3 ± 2	0	0
Fertilization (%)	2775/3829 (72.5)	5072/7126 (71.1)	6418/12,220 (52.5)^c^	2477/5059 (49.0)^c^
Clinical pregnancy (%)	221 (60.7)^b^	353 (45.5)^b^	500 (43.2)^d^	187 (33.7)^d^

^a^Student’s *t*-test, two independent samples, effect of epididymal cryopreservation on sperm motility, *P* < 0.0001; ^b^*χ*^2^, 2 × 2, 1 *df*, effect of epididymal cryopreservation on clinical pregnancy rate, *P* < 0.0001; ^c^*χ*^2^, 2 × 2, 1 *df*, effect of testicular cryopreservation on fertilization rate, *P* = 0.02; ^d^*χ*^2^, 2 × 2, 1 *df*, effect of testicular cryopreservation on clinical pregnancy rate, *P* < 0.0001.

Overall, the implementation of surgical retrievals in conjunction with ICSI cycles has aided many men with no chance of natural conception to have their own child. However, further observation and follow-up studies need to be carried out as the cohort of reproductive aged offspring from these procedures increases.

### Extreme ICSI cases

**Figure 5:**
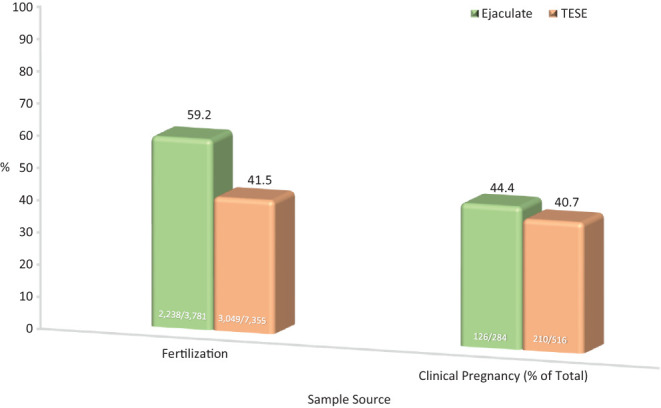
Comparison of fertilization and clinical pregnancy rates in cases with few spermatozoa identified. In 986 cycles, extremely few spermatozoa were seen after high-speed centrifugation. Samples were searched for injectable spermatozoa in drops under oil for up to several hours by multiple embryologists until all oocytes were injected. Oocytes injected with ejaculate spermatozoa demonstrated a higher fertilization rate compared to those injected with testicular specimen (*χ*^2^, 2 × 2, 1 *df*; *P* < 0.0001). Clinical pregnancy rates remained comparable between the two sperm sources.

When very few to no spermatozoa are seen even after high-speed centrifugation, an extensive search is performed prior to ICSI in order to identify cells for injection. In 986 ICSI cycles, as updated from Palermo *et al*. (Palermo *et al*. 2014*d*), a search for injectable spermatozoa that required at least 30 min was carried out in ejaculate and testicular biopsy specimens. Ejaculated spermatozoa demonstrated a significantly higher fertilization rate in comparison to TESE specimen without however, affecting clinical pregnancy rates, which remained comparable between the two sources (Fig. 5). A similar study of patients with virtual azoospermia found an improved clinical pregnancy and implantation rate when using surgical vs ejaculated specimen (Ketabchi 2016).

Although scant spermatozoa can occasionally be found in ejaculated and testicular samples of these aforementioned cases, 40–60% of NOA patients that undergo a TESE/micro-TESE fail to retrieve spermatozoa (Bernie *et al*. 2013, Vloeberghs *et al*. 2015). The high occurrence of failed TESEs in NOA patients has prompted research into alternative approaches for these men to conceive a child of their own. In the event that no spermatozoa are identified in a testicular sample, studies have reported that in patients identified as having scant spermatogenesis and even maturation arrest, the use of round spermatids for injection (ROSI) has the ability to fertilize an oocyte (Vanderzwalmen *et al*. 1997). It should be noted however, that this procedure has incited vehement assertion that cases where round spermatids are identified always contain elongating spermatids as well (Silber *et al*. 2000). Nevertheless, a recent study using ROSI has now claimed to have yielded 14 babies as of 2015 from a patient cohort that failed their first micro-TESE (Tanaka *et al*. 2015). Although these data seem encouraging regarding patients that did not appear to have spermatozoa upon surgical extraction and further evaluation, injection of these round cells did not consistently induce oocyte activation without the aid of electric stimulation. From what we have learned thus far about the role of paternal RNAs in embryogenesis, these observations could indicate a lack of necessary transcripts available to the oocyte once a round spermatid is injected, possibly due to its spermiogenic block. As a result, close follow-ups of the offspring should be conducted before any clinical value can be assumed from the procedure.

## ICSI safety

The issues related to the safety of intracytoplasmic sperm injection will be discussed in further detail following this chapter.

The general adoption of ICSI and its success has not been without some concern that this procedure bypasses natural sperm selection (Cummins & Jequier 1994, de Kretser 1995, De Rycke *et al*. 2002, Edwards & Ludwig 2003) bringing into question the growth, cognitive development and postnatal well-being of the offspring as well as the impact on their future reproductive capacity (Bowen *et al*. 1998, Schieve *et al*. 2004).

Several surveys of children born through ART evidenced an increased rate of neonatal malformations (Hansen *et al*. 2002), lower birth weights (Schieve *et al*. 2002), the prevalence of imprinting errors (DeBaun *et al*. 2003, Gicquel *et al*. 2003, Maher *et al*. 2003, Orstavik 2003, Halliday *et al*. 2004), and even some forms of cancer (Moll *et al*. 2003). However, these studies did not link the cases of imprinting disorders or childhood neoplasia to the ICSI procedure itself (Edwards & Ludwig 2003).

Nevertheless, concerns related to the utilization of these less than ideal spermatozoa may still be the reason for genetic and congenital abnormalities (Ludwig & Katalinic 2002). Contrary to these concerns, we observed that the rate of malformation from ICSI was no higher than naturally conceived offspring reported in New York State (Palermo *et al*. 1996*b*). Additionally, a study of 14,211 ART children determined that the malformation rate with ICSI is comparable to that with IVF (Palermo *et al*. 2013).

In fact, the follow-up literature assessing ICSI offspring from neonates to adolescents have shown satisfactory physical and psychological development (Belva *et al*. 2007, 2011, 2012, Basatemur & Sutcliffe 2008, Knoester *et al*. 2008, Leunens *et al*. 2008, Goldbeck *et al*. 2009, Basatemur *et al*. 2010, Carson *et al*. 2013).

Among all epigenetic diseases, Beckwith–Wiedemann Syndrome (BWS) is the only disorder that has been unequivocally linked to ART (Sutcliffe *et al*. 2006) although not related to a specific reproductive technique. A more recent study assessed the methylome in cord blood of children generated through standard *in vitro* insemination, ICSI, and compared to a naturally conceived offspring (El Hajj *et al*. 2017). The initial analysis failed to evidence any particular effect of the ART on the DNA methylation patterns. A confirmatory analysis pointed at some small differential methylation of two specific genes (*ATG4C* and *SNORD114*). However, in consideration of several confounding factors, the authors were not able to distinguish between the effect of a specific insemination method or the contribution of a male factor. The authors also acknowledged that this study, carried out by a non-clinically validated assay, appears inconclusive until a larger study is carried out. Separately from the insemination procedure, long-term blastocyst culture has been associated with gene expression imbalances (Basatemur & Sutcliffe 2008, Rivera *et al*. 2008), furthermore, there is presently no clear evidence that resort to ICSI predisposes offspring to gene expression disorders, in animals or humans (Wilson *et al*. 2007).

In summary, the most prevalent factor that may contribute to adverse postnatal outcomes in children conceived by ART stems from high order-gestation (Pereira *et al*. 2016*a*), a common consequence of assisted reproduction. Indeed, the adoption of single embryo transfer procedures has helped to diminish this issue. Additional complications such as prematurity, low birth weight, perinatal mortality, as well as congenital malformations have been indiscriminately linked to ART, though the primary factor responsible appears to be inherent in the infertility indication itself. While ICSI is not a causation of long-term neurodevelopmental defects or cancer, further follow-up studies into adulthood should be continued to better inform our understanding of assisted reproduction and more comprehensively answer these questions (Palermo *et al*. 2013).

In consideration of the foregoing, the first studies on the oldest ICSI cohort have been recently published, assessing the male and female offspring independently compared to a naturally conceived control. A survey of male reproductive hormones in young ICSI men proved to be comparable to naturally conceived peers (Belva *et al*. 2017*a*). A second study in a similar cohort of ICSI men focused assessing their spermatogenesis by measuring the semen characteristics in comparison to a control. This study demonstrated lower semen parameters in the ICSI cohort, although all metrics were still considered above threshold according to the WHO criteria (WHO 2010, Belva *et al*. 2016). The fact that these ICSI offspring have semen parameters within the normal range, and therefore supports the notion of their potential ability to conceive, is somewhat reassuring, considering they were generated from fathers afflicted by male infertility incapable of procreating naturally or by standard *in vitro* insemination. Alike, the most recent follow-up study on young women born from ICSI has shown encouraging results in relation to their fertility status, demonstrating a comparable hormonal profile to naturally conceived girls (Belva *et al*. 2017*b*). Nonetheless, as these cohorts assessed are relatively small, further multicenter studies would be welcome in order to confirm these preliminary findings.

## Conclusions

In spite of the fact that ICSI was developed almost by chance, and earlier conclusions that the intracytoplasmic approach was too invasive and unreliable, its value has been affirmed in a variety of challenging situations, particularly for severe male factor couples wishing to have their own genetic child. Not least, over these last 25 years, ICSI has made possible the utilization of immature forms of the male gamete such as epididymal and testicular spermatozoa.

At our center, ICSI is additionally used in all cases using cryopreserved donor and husband spermatozoa samples in order to compensate for poor survival upon thawing. The utilization of a single spermatozoon has been instrumental to permit proper fertilization of oocytes prior to cryopreservation and in cases with a low egg yield, as often seen in couples with advanced maternal age or in poor responders to ovarian superovulation.

The advantages of an insemination technique that leveled the equivalency between a single male and female gamete have been indispensable in helping to explain specific aspects of sperm–oocyte interaction, such as understanding the acrosomal function in relation to the stability of the spermiolemma and its inherent connection to sperm motility as well as validating the mechanism of inheritance of the sperm centrosome.

Failure to achieve fertilization with ICSI has stimulated research into the mechanisms behind oocyte activation and has conversely indicated that a dysmature ooplasm is not receptive even to a fully competent spermatozoon.

The introduction of assays, particularly the assessment of chromatin integrity of sperm cells has evidenced that DNA fragmentation can influence the reproductive outcome of couples, running the gamut of assisted reproductive techniques from programmed intercourse and IUI, to standard *in vitro* insemination, and rarely intracytoplasmic injection.

It appears that ICSI will continue to play a role in the immediate and distant future of assisted reproductive technology and remain of paramount importance in cases involving mitochondrial therapies of micro-manipulated oocytes or eventually, for use of sperm cells generated through *in vitro* spermatogenesis and even neo-gametogenesis from stem cells.

## Declaration of interest

The authors declare that there is no conflict of interest that could be perceived as prejudicing the impartiality of this review.

## Funding

This research did not receive any specific grant from any funding agency in the public, commercial or not-for-profit sector.

## References

[bib1] Aitken RJ De Iuliis GN McLachlan RI 2009 Biological and clinical significance of DNA damage in the male germ line. International Journal of Andrology 32 46–56. (10.1111/j.1365-2605.2008.00943.x) 19076252

[bib2] Asada H Sueoka K Hashiba T Kuroshima M Kobayashi N Yoshimura Y 2000 The effects of age and abnormal sperm count on the nondisjunction of spermatozoa. Journal of Assisted Reproduction and Genetics 17 51–59. (10.1023/A:1009454114973) 10754784 PMC3455190

[bib3] Baccetti B Burrini AG Collodel G Magnano AR Piomboni P Renieri T Sensini C 1989 Morphogenesis of the decapitated and decaudated sperm defect in two brothers. Gamete Research 23 181–188. (10.1002/mrd.1120230205) 2731903

[bib4] Barros A Sousa M Oliveira C Silva J Almeida V Beires J 1997 Pregnancy and birth after intracytoplasmic sperm injection with totally immotile sperm recovered from the ejaculate. Fertility and Sterility 67 1091–1094. (10.1016/S0015-0282(97)81444-6) 9176449

[bib5] Barthelemy C Tharanne MJ Lebos C Lecomte P Lansac J 1990 Tail stump spermatozoa: morphogenesis of the defect. An ultrastructural study of sperm and testicular biopsy. Andrologia 22 417–425. (10.1111/j.1439-0272.1990.tb02020.x) 2073052

[bib6] Basatemur E Sutcliffe A 2008 Follow-up of children born after ART. Placenta 29 (Supplement B) 135–140. (10.1016/j.placenta.2008.08.013) 18790325

[bib7] Basatemur E Shevlin M Sutcliffe A 2010 Growth of children conceived by IVF and ICSI up to 12 years of age. Reproductive BioMedicine Online 20 144–149. (10.1016/j.rbmo.2009.10.006) 20159000

[bib8] Bedford JM Bent MJ Calvin H 1973 Variations in the structural character and stability of the nuclear chromatin in morphologically normal human spermatozoa. Journal of Reproduction and Fertility 33 19–29. (10.1530/jrf.0.0330019) 4573026

[bib9] Belva F Henriet S Liebaers I Van Steirteghem A Celestin-Westreich S Bonduelle M 2007 Medical outcome of 8-year-old singleton ICSI children (born >or=32 weeks’ gestation) and a spontaneously conceived comparison group. Human Reproduction 22 506–515. (10.1093/humrep/del372) 16982659

[bib10] Belva F Bonduelle M Schiettecatte J Tournaye H Painter RC Devroey P De Schepper J 2011 Salivary testosterone concentrations in pubertal ICSI boys compared with spontaneously conceived boys. Human Reproduction 26 438–441. (10.1093/humrep/deq345) 21138905

[bib11] Belva F Roelants M Painter R Bonduelle M Devroey P De Schepper J 2012 Pubertal development in ICSI children. Human Reproduction 27 1156–1161. (10.1093/humrep/des001) 22328555

[bib12] Belva F Bonduelle M Roelants M Michielsen D Van Steirteghem A Verheyen G Tournaye H 2016 Semen quality of young adult ICSI offspring: the first results. Human Reproduction 31 2811–2820. (10.1093/humrep/dew245) 27707840

[bib13] Belva F Roelants M De Schepper J Van Steirteghem A Tournaye H Bonduelle M 2017a Reproductive hormones of ICSI-conceived young adult men: the first results. Human Reproduction 32 439–446. (10.1093/humrep/dew324) 28007789

[bib14] Belva F Roelants M Vloeberghs V Schiettecatte J Evenepoel J Bonduelle M de Vos M 2017b Serum reproductive hormone levels and ultrasound findings in female offspring after intracytoplasmic sperm injection: first results. Fertility and Sterility 107 934–939. (10.1016/j.fertnstert.2017.02.102) 28292621

[bib15] Benagiano G Gianaroli L 2004 The new Italian IVF legislation. Reproductive BioMedicine Online 9 117–125. (10.1016/S1472-6483(10)62118-9) 15333237

[bib16] Bendikson KA Neri QV Takeuchi T Toschi M Schlegel PN Rosenwaks Z Palermo GD 2008 The outcome of intracytoplasmic sperm injection using occasional spermatozoa in the ejaculate of men with spermatogenic failure. Journal of Urology 180 1060–1064. (10.1016/j.juro.2008.05.025) 18639294

[bib17] Bernie AM Ramasamy R Schlegel PN 2013 Predictive factors of successful microdissection testicular sperm extraction. Basic and Clinical Andrology 23 5. (10.1186/2051-4190-23-5) 25763186 PMC4346292

[bib18] Biermann K Steger K 2007 Epigenetics in male germ cells. Journal of Andrology 28 466–480. (10.2164/jandrol.106.002048) 17287457

[bib19] Boerke A Dieleman SJ Gadella BM 2007 A possible role for sperm RNA in early embryo development. Theriogenology 68 (Supplement 1) S147–S155. (10.1016/j.theriogenology.2007.05.058) 17583784

[bib20] Bosch M Rajmil O Egozcue J Templado C 2003 Linear increase of structural and numerical chromosome 9 abnormalities in human sperm regarding age. European Journal of Human Genetics 11 754–759. (10.1038/sj.ejhg.5201049) 14512965

[bib21] Boulet SL Mehta A Kissin DM Warner L Kawwass JF Jamieson DJ 2015 Trends in use of and reproductive outcomes associated with intracytoplasmic sperm injection. JAMA 313 255–263. (10.1001/jama.2014.17985) 25602996 PMC4343214

[bib22] Bowen JR Gibson FL Leslie GI Saunders DM 1998 Medical and developmental outcome at 1 year for children conceived by intracytoplasmic sperm injection. Lancet 351 1529–1534. (10.1016/S0140-6736(98)10168-X) 10326534

[bib23] Brewer L Corzett M Balhorn R 2002 Condensation of DNA by spermatid basic nuclear proteins. Journal of Biological Chemistry 277 38895–38900. (10.1074/jbc.M204755200) 12140285

[bib24] Carrell DT 2012 Epigenetics of the male gamete. Fertility and Sterility 97 267–274. (10.1016/j.fertnstert.2011.12.036) 22289286

[bib25] Carson C Sacker A Kelly Y Redshaw M Kurinczuk JJ Quigley MA 2013 Asthma in children born after infertility treatment: findings from the UK Millennium Cohort Study. Human Reproduction 28 471–479. (10.1093/humrep/des398) 23223378 PMC3545639

[bib26] Chen C Hu JC Neri QV Rosenwaks Z Palermo GD 2011 Kinetic characteristics and DNA integrity of human spermatozoa. In Abstracts of the 20th Annual Meeting of the European Society of Human Reproduction and Embryology, Berlin, Germany, 27–30 June 2004. Human Reproduction 19 (Supplement 1).

[bib27] Chi HJ Koo JJ Song SJ Lee JY Chang SS 2004 Successful fertilization and pregnancy after intracytoplasmic sperm injection and oocyte activation with calcium ionophore in a normozoospermic patient with extremely low fertilization rates in intracytoplasmic sperm injection cycles. Fertility and Sterility 82 475–477. (10.1016/j.fertnstert.2004.01.038) 15302306

[bib28] Chohan KR Griffin JT Lafromboise M De Jonge CJ Carrell DT 2006 Comparison of chromatin assays for DNA fragmentation evaluation in human sperm. Journal of Andrology 27 53–59. (10.2164/jandrol.05068) 16400078

[bib29] Cissen M van Wely M Scholten I Mansell S de Bruin JP Mol BW Braat D Repping S Hamer G 2016 Measuring sperm DNA fragmentation and clinical outcomes of medically assisted reproduction: a systematic review and meta-analysis. PLoS ONE 11 e0165125. (10.1371/journal.pone.0165125) 27832085 PMC5104467

[bib30] Cohen J Malter H Fehilly C Wright G Elsner C Kort H Massey J 1988 Implantation of embryos after partial opening of oocyte zona pellucida to facilitate sperm penetration. Lancet 2 162. (10.1016/S0140-6736(88)90710-6) 2899210

[bib31] Colombero LT Hariprashad JJ Tsai MC Rosenwaks Z Palermo GD 1999a Incidence of sperm aneuploidy in relation to semen characteristics and assisted reproductive outcome. Fertility and Sterility 72 90–96. (10.1016/S0015-0282(99)00158-2) 10428154

[bib32] Colombero LT Takeuchi T Sills ES Breed WG Rosenwaks Z Palermo GD 1999b A comparison of human spermatozoa immunolabeling features using xenogenic reagents for centrosomal proteins. Clinical and Experimental Obstetrics and Gynecology 26 141–146. 10668138

[bib33] Cooper TG Neuwinger J Bahrs S Nieschlag E 1992 Internal quality control of semen analysis. Fertility and Sterility 58 172–178. (10.1016/S0015-0282(16)55156-5) 1624001

[bib34] Cummins JM Jequier AM 1994 Treating male infertility needs more clinical andrology, not less. Human Reproduction 9 1214–1219. (10.1093/oxfordjournals.humrep.a138681) 7962420

[bib35] Dadoune J-P 2003 Expression of mammalian spermatozoal nucleoproteins. Microscopy Research and Technique 61 56–75. (10.1002/jemt.10317) 12672123

[bib36] Dadoune JP 2009 Spermatozoal RNAs: what about their functions? Microscopy Research and Technique 72 536–551. (10.1002/jemt.20697) 19283828

[bib37] de Kretser DM 1995 The potential of intracytoplasmic sperm injection (ICSI) to transmit genetic defects causing male infertility. Reproduction, Fertility, and Development 7 137–141; discussion 141–132. (10.1071/RD9950137) 7480831

[bib38] De Rycke M Liebaers I Van Steirteghem A 2002 Epigenetic risks related to assisted reproductive technologies: risk analysis and epigenetic inheritance. Human Reproduction 17 2487–2494. (10.1093/humrep/17.10.2487) 12351517

[bib39] DeBaun MR Niemitz EL Feinberg AP 2003 Association of in vitro fertilization with Beckwith-Wiedemann syndrome and epigenetic alterations of LIT1 and H19. American Journal of Human Genetics 72 156–160. (10.1086/346031) 12439823 PMC378620

[bib40] Donate A Estop AM Giraldo J Templado C 2016 Paternal age and numerical chromosome abnormalities in human spermatozoa. Cytogenetic and Genome Research 148 241–248. (10.1159/000446724) 27322585

[bib41] Dyer S Chambers GM de Mouzon J Nygren KG Zegers-Hochschild F Mansour R Ishihara O Banker M Adamson GD 2016 International Committee for Monitoring Assisted Reproductive Technologies world report: Assisted Reproductive Technology 2008, 2009 and 2010. Human Reproduction 31 1588–1609. (10.1093/humrep/dew082) 27207175

[bib42] Edwards RG Ludwig M 2003 Are major defects in children conceived in vitro due to innate problems in patients or to induced genetic damage? Reproductive BioMedicine Online 7 131–138. (10.1016/S1472-6483(10)61742-7) 14567877

[bib43] El Hajj N Haertle L Dittrich M Denk S Lehnen H Hahn T Schorsch M Haaf T 2017 DNA methylation signatures in cord blood of ICSI children. Human Reproduction 32 1761–1769. (10.1093/humrep/dex209) 28575352 PMC5850272

[bib44] ESHRE 2012 World’s number of IVF and ICSI babies has now reached a calculated total of 5 million. ScienceDaily 2 July 2012. (www.sciencedaily.com/releases/2012/07/120702134746.htm)

[bib45] Esteves SC Sanchez-Martin F Sanchez-Martin P Schneider DT Gosalvez J 2015 Comparison of reproductive outcome in oligozoospermic men with high sperm DNA fragmentation undergoing intracytoplasmic sperm injection with ejaculated and testicular sperm. Fertility and Sterility 104 1398–1405. (10.1016/j.fertnstert.2015.08.028) 26428305

[bib46] Evenson D Wixon R 2006 Meta-analysis of sperm DNA fragmentation using the sperm chromatin structure assay. Reproductive BioMedicine Online 12 466–472. (10.1016/S1472-6483(10)62000-7) 16740220

[bib47] Flaherty SP Dianna P Swann NJ Matthews CD 1995 Aetiology of failed and abnormal fertilization after intracytoplasmic sperm injection1. Human Reproduction 10 2623–2629. (10.1093/oxfordjournals.humrep.a135757) 8567782

[bib48] Funaro M Paduch DA 2014 Novel markers of male infertility. Methods in Molecular Biology 1154 233–250. (10.1007/978-1-4939-0659-8_9) 24782011

[bib49] Furrow RE Christiansen FB Feldman MW 2011 Environment-sensitive epigenetics and the heritability of complex diseases. Genetics 189 1377–1387. (10.1534/genetics.111.131912) 21968193 PMC3241426

[bib50] Gambera L Falcone P Mencaglia L Collodel G Serafini F De Leo V Piomboni P 2009 Intracytoplasmic sperm injection and pregnancy with decapitated sperm. Fertility and Sterility 93 1347.e1347–1347.e1312. (10.1016/j.fertnstert.2008.12.087) 19233351

[bib51] Garcia-Herrero S Meseguer M Martinez-Conejero JA Remohi J Pellicer A Garrido N 2010 The transcriptome of spermatozoa used in homologous intrauterine insemination varies considerably between samples that achieve pregnancy and those that do not. Fertility and Sterility 94 1360–1373. (10.1016/j.fertnstert.2009.07.1671) 19796764

[bib52] Garrido N Martinez-Conejero JA Jauregui J Horcajadas JA Simon C Remohi J Meseguer M 2009 Microarray analysis in sperm from fertile and infertile men without basic sperm analysis abnormalities reveals a significantly different transcriptome. Fertility and Sterility 91 1307–1310. (10.1016/j.fertnstert.2008.01.078) 18367176

[bib53] Gatimel N Parinaud J Leandri RD 2016 Intracytoplasmic morphologically selected sperm injection (IMSI) does not improve outcome in patients with two successive IVF-ICSI failures. Journal of Assisted Reproduction and Genetics 33 349–355. (10.1007/s10815-015-0645-5) 26754750 PMC4785160

[bib54] Gicquel C Gaston V Mandelbaum J Siffroi JP Flahault A Le Bouc Y 2003 In vitro fertilization may increase the risk of Beckwith-Wiedemann syndrome related to the abnormal imprinting of the KCN1OT gene. American Journal of Human Genetics 72 1338–1341. (10.1086/374824) 12772698 PMC1180288

[bib55] Girard A Sachidanandam R Hannon GJ Carmell MA 2006 A germline-specific class of small RNAs binds mammalian Piwi proteins. Nature 442 199–202. (10.1038/nature04917) 16751776

[bib56] Glazener CM Coulson C Lambert PA Watt EM Hinton RA Kelly NJ Hull MG 1987 The value of artificial insemination with husband’s semen in infertility due to failure of postcoital sperm-mucus penetration – controlled trial of treatment. British Journal of Obstetrics and Gynaecology 94 774–778. (10.1111/j.1471-0528.1987.tb03725.x) 3311134

[bib57] Goldbeck L Gagsteiger F Mindermann I Strobele S Izat Y 2009 Cognitive development of singletons conceived by intracytoplasmic sperm injection or in vitro fertilization at age 5 and 10 years. Journal of Pediatric Psychology 34 774–781. (10.1093/jpepsy/jsn120) 19036784

[bib58] Gordon JW Talansky BE 1986 Assisted fertilization by zona drilling: a mouse model for correction of oligospermia. Journal of Experimental Zoology 239 347–354. (10.1002/jez.1402390306) 3760806

[bib59] Gordon JW Grunfeld L Garrisi GJ Talansky BE Richards C Laufer N 1988 Fertilization of human oocytes by sperm from infertile males after zona pellucida drilling. Fertility and Sterility 50 68–73. (10.1016/S0015-0282(16)60010-9) 3384120

[bib60] Griffin DK Abruzzo MA Millie EA Feingold E Hassold TJ 1996 Sex ratio in normal and disomic sperm: evidence that the extra chromosome 21 preferentially segregates with the Y chromosome. American Journal of Human Genetics 59 1108–1113. 8900240 PMC1914829

[bib61] Grivna ST Pyhtila B Lin H 2006 MIWI associates with translational machinery and PIWI-interacting RNAs (piRNAs) in regulating spermatogenesis. PNAS 103 13415–13420. (10.1073/pnas.0605506103) 16938833 PMC1569178

[bib62] Habermann H Seo R Cieslak J Niederberger C Prins GS Ross L 2000 In vitro fertilization outcomes after intracytoplasmic sperm injection with fresh or frozen-thawed testicular spermatozoa. Fertility and Sterility 73 955–960. (10.1016/S0015-0282(00)00416-7) 10785220

[bib63] Hachem A Godwin J Ruas M Lee HC Ferrer Buitrago M Ardestani G Bassett A Fox S Navarrete F de Sutter P 2017 PLCζ is the physiological trigger of the Ca^2+^ oscillations that induce embryogenesis in mammals but conception can occur in its absence. Development 144 2914-2924. (10.1242/dev.150227) 28694258 PMC5592814

[bib64] Halliday J Oke K Breheny S Algar E J Amor D 2004 Beckwith-Wiedemann syndrome and IVF: a case-control study. American Journal of Human Genetics 75 526–528. (10.1086/423902) 15284956 PMC1182036

[bib65] Hamada AJ Esteves SC Agarwal A 2013 A comprehensive review of genetics and genetic testing in azoospermia. Clinics 68 39–60. (10.6061/clinics/2013(Sup01)06) PMC3583155 23503954

[bib66] Hamatani T 2012 Human spermatozoal RNAs. Fertility and Sterility 97 275–281. (10.1016/j.fertnstert.2011.12.035) 22289287

[bib67] Hammoud S Liu L Carrell DT 2009a Protamine ratio and the level of histone retention in sperm selected from a density gradient preparation. Andrologia 41 88–94. (10.1111/j.1439-0272.2008.00890.x) 19260844

[bib68] Hammoud SS Nix DA Zhang H Purwar J Carrell DT Cairns BR 2009b Distinctive chromatin in human sperm packages genes for embryo development. Nature 460 473–478. (10.1038/nature08162) 19525931 PMC2858064

[bib69] Hansen M Kurinczuk JJ Bower C Webb S 2002 The risk of major birth defects after intracytoplasmic sperm injection and in vitro fertilization. New England Journal of Medicine 346 725–730. (10.1056/NEJMoa010035) 11882727

[bib70] Hassold T Hunt P 2001 To err (meiotically) is human: the genesis of human aneuploidy. Nature Reviews Genetics 2 280–291. (10.1038/35066065) 11283700

[bib71] Hu JCY Monahan D Neri QV Rosenwaks Z Palermo GD 2011 The role of sperm aneuploidy assay. Fertility and Sterility 96 S24–S25. (10.1016/j.fertnstert.2011.07.102)

[bib72] Irvine DS Aitken RJ Lees MM Reid C 1986 Failure of high intrauterine insemination of husband’s semen. Lancet 2 972–973. (10.1016/S0140-6736(86)90621-5) 2877148

[bib73] Jodar M Selvaraju S Sendler E Diamond MP Krawetz SA 2013 The presence, role and clinical use of spermatozoal RNAs. Human Reproduction Update 19 604–624. (10.1093/humupd/dmbib31) 23856356 PMC3796946

[bib74] Jodar M Sendler E Moskovtsev SI Librach CL Goodrich R Swanson S Hauser R Diamond MP Krawetz SA 2015 Absence of sperm RNA elements correlates with idopathic male infertility. Science Translational Medicine 7 295re296. (10.1126/scitranslmed.aab1287) PMC4721635 26157032

[bib75] Johnson MH 1989 The effect on fertilization of exposure of mouse oocytes to dimethyl sulfoxide: an optimal protocol. Journal of In Vitro Fertilization and Embryo Transfer 6 7. (10.1007/BF01134574) 2677190

[bib76] Junca AM Dumont M Cornet D Douard S De Mouzon J Prisant N 2010 Is intracytoplasmic morphologically selected sperm injection (IMSI) detrimental for pregnancy outcome? Fertility and Sterility 94 S31. (10.1016/j.fertnstert.2010.07.118)

[bib77] Kacem O Sifer C Barraud-Lange V Ducot B De Ziegler D Poirot C Wolf J 2010 Sperm nuclear vacuoles, as assessed by motile sperm organellar morphological examination, are mostly of acrosomal origin. Reproductive BioMedicine Online 20 132–137. (10.1016/j.rbmo.2009.10.014) 20158998

[bib78] Kahraman S Özgür S Alataş C Aksoy S Balaban B Evrenkaya T Nuhoğlu A Taşdemir M Biberoğlu K Schoysman R 1996 High implantation and pregnancy rates with testicular sperm extraction and intracytoplasmic sperm injection in obstructive and non-obstructive azoospermia. Human Reproduction 11 673–676. (10.1093/HUMREP/11.3.673) 8671290

[bib79] Kashir J Heindryckx B Jones C De Sutter P Parrington J Coward K 2010 Oocyte activation, phospholipase C zeta and human infertility. Human Reproduction Update 16 690–703. (10.1093/humupd/dmq018) 20573804

[bib80] Ketabchi AA 2016 Intracytoplasmic sperm injection outcomes with freshly ejaculated sperms and testicular or epididymal sperm extraction in patients with idiopathic cryptozoospermia. Nephro-Urology Monthly 8 e41375. (10.5812/numonthly.41375) 27896242 PMC5120410

[bib81] Kiessling AA Loutradis D McShane PM Jackson KV 1988 Fertilization in trypsin-treated oocytes. Annals of the New York Academy of Sciences 541 614–620. (10.1111/j.1749-6632.1988.tb22298.x) 3195940

[bib82] Kim VN 2006 Small RNAs just got bigger: Piwi-interacting RNAs (piRNAs) in mammalian testes. Genes and Development 20 1993–1997. (10.1101/gad.1456106) 16882976

[bib83] Kimble M Kuriyama R 1992 Functional components of microtubule-organizing centers. International Review of Cytology 136 1–50. (10.1016/s0074-7696(08)62049-5) 1506143

[bib84] Knoester M Helmerhorst FM Vandenbroucke JP van der Westerlaken LA Walther FJ Veen S 2008 Perinatal outcome, health, growth & medical care utilization of 5- to 8-year-old intracytoplasmic sperm injection singletons. Fertility and Sterility 89 1133–1146. (10.1016/j.fertnstert.2007.04.049) 18177652

[bib85] Krawetz SA Kruger A Lalancette C Tagett R Anton E Draghici S Diamond MP 2011 A survey of small RNAs in human sperm. Human Reproduction 26 3401–3412. (10.1093/humrep/der329) 21989093 PMC3212879

[bib86] Kruger TF Menkveld R Stander FSH Lombard CJ Van der Merwe JP van Zyl JA Smith K 1986 Sperm morphologic features as a prognostic factor in in vitro fertilization. Fertility and Sterility 46 1118–1123. (10.1016/S0015-0282(16)49891-2) 2946611

[bib87] Kumar G Patel D Naz RK 1993 c-MYC mRNA is present in human sperm cells. Cellular and Molecular Biology Research 39 111–117. 8220581

[bib88] Kumar K Deka D Singh A Mitra DK Vanitha BR Dada R 2012 Predictive value of DNA integrity analysis in idiopathic recurrent pregnancy loss following spontaneous conception. Journal of Assisted Reproduction and Genetics 29 861–867. (10.1007/s10815-012-9801-3) 22692280 PMC3463671

[bib89] Künzle R Mueller MD Hänggi W Birkhäuser MH Drescher H Bersinger NA 2003 Semen quality of male smokers and nonsmokers in infertile couples. Fertility and Sterility 79 287–291. (10.1016/S0015-0282(02)04664-2) 12568836

[bib90] Laws-King A Trounson A Sathananthan H Kola I 1987 Fertilization of human oocytes by microinjection of a single spermatozoon under the zona pellucida. Fertility and Sterility 48 637–642. (10.1016/S0015-0282(16)59478-3) 3653422

[bib91] Leunens L Celestin-Westreich S Bonduelle M Liebaers I Ponjaert-Kristoffersen I 2008 Follow-up of cognitive and motor development of 10-year-old singleton children born after ICSI compared with spontaneously conceived children. Human Reproduction 23 105–111. (10.1093/humrep/dem257) 17981820

[bib92] Levine BA Feinstein J Neri QV Goldschlag D Rosenwaks Z Belongie S Palermo GD 2015 Three-dimensional sperm surface reconstruction: a novel approach to assessing sperm morphology. Fertility and Sterility 104 e14–e15. (10.1016/j.fertnstert.2015.08.024) 26363386

[bib93] Lewis SEM John Aitken R Conner SJ Iuliis GD Evenson DP Henkel R Giwercman A Gharagozloo P 2013 The impact of sperm DNA damage in assisted conception and beyond: recent advances in diagnosis and treatment. Reproductive BioMedicine Online 27 325–337. (10.1016/j.rbmo.2013.06.014) 23948450

[bib94] Lewis-Jones I Aziz N Seshadri S Douglas A Howard P 2003 Sperm chromosomal abnormalities are linked to sperm morphologic deformities. Fertility and Sterility 79 212–215. (10.1016/S0015-0282(02)04411-4) 12524092

[bib95] Lo Monte G Murisier F Piva I Germond M Marci R 2013 Focus on intracytoplasmic morphologically selected sperm injection (IMSI): a mini-review. Asian Journal of Andrology 15 608–615. (10.1038/aja.2013.54) 23832017 PMC3881647

[bib96] Ludwig M Diedrich K 1999 Regulation of assisted reproductive technology: the German experience. In Regulation of Assisted Reproductive Technology: The German Experience, p 3. Ed PRBrinsden. New York: Parthenon Publishing Group Inc.

[bib97] Ludwig M Katalinic A 2002 Malformation rate in fetuses and children conceived after ICSI: results of a prospective cohort study. Reproductive BioMedicine Online 5 171–178. (10.1016/S1472-6483(10)61621-5) 12419043

[bib98] MacLeod J Wang Y 1979 Male fertility potential in terms of semen quality: a review of the past, a study of the present. Fertility and Sterility 31 103–116. (10.1016/S0015-0282(16)43808-2) 367823

[bib99] Maher ER Brueton LA Bowdin SC Luharia A Cooper W Cole TR Macdonald F Sampson JR Barratt CL Reik W 2003 Beckwith-Wiedemann syndrome and assisted reproduction technology (ART). Journal of Medical Genetics 40 62–64. (10.1136/jmg.40.1.62) 12525545 PMC1735252

[bib100] Mallidis C Howard EJ Baker HW 1991 Variation of semen quality in normal men. International Journal of Andrology 14 99–107. (10.1111/j.1365-2605.1991.tb01071.x) 1869320

[bib101] Mangoli VS Dandekar SP Desai SK Mangoli RV 2008 The outcome of ART in males with impaired spermatogenesis. Journal of Human Reproductive Sciences 1 73–76. (10.4103/0974-1208.44114) 19562049 PMC2700665

[bib102] Martin RH 2006 Meiotic chromosome abnormalities in human spermatogenesis. Reproductive Toxicology 22 142–147. (10.1016/j.reprotox.2006.03.013) 16714098

[bib103] Martin RH Spriggs E Ko E Rademaker AW 1995 The relationship between paternal age, sex ratios, and aneuploidy frequencies in human sperm, as assessed by multicolor FISH. American Journal of Human Genetics 57 1395–1399. 8533769 PMC1801415

[bib104] Mathur PP D’Cruz SC 2011 The effect of environmental contaminants on testicular function. Asian Journal of Andrology 13 585–591. (10.1038/aja.2011.40) 21706039 PMC3739630

[bib105] Matson PL 1995 External quality assessment for semen analysis and sperm antibody detection: results of a pilot scheme. Human Reproduction 10 620–625. (10.1093/oxfordjournals.humrep.a135999) 7782442

[bib106] McCoy RC Demko Z Ryan A Banjevic M Hill M Sigurjonsson S Rabinowitz M Fraser HB Petrov DA 2015 Common variants spanning PLK4 are associated with mitotic-origin aneuploidy in human embryos. Science 348 235–238. (10.1126/science.aaa3337) 25859044 PMC5519344

[bib107] McIntosh GC Olshan AF Baird PA 1995 Paternal age and the risk of birth defects in offspring. Epidemiology 6 282–288. (10.1097/00001648-199505000-00016) 7619937

[bib108] McIver SC Roman SD Nixon B EA McLaughlin 2012 miRNA and mammalian male germ cells. Human Reproduction Update 18 44–59. (10.1093/humupd/dmr041) 21989172

[bib109] Mencaglia L Falcone P Lentini GM Consigli S Pisoni M Lofiego V Guidetti R Piomboni P De Leo V 2005 ICSI for treatment of human immunodeficiency virus and hepatitis C virus-serodiscordant couples with infected male partner. Human Reproduction 20 2242–2246. (10.1093/humrep/debib31) 15946998

[bib110] Meseguer M Santiso R Garrido N García-Herrero S Remohí J Fernandez JL 2011 Effect of sperm DNA fragmentation on pregnancy outcome depends on oocyte quality. Fertility and Sterility 95 124–128. (10.1016/j.fertnstert.2010.05.055) 20643402

[bib111] Miller D Ostermeier GC 2006 Towards a better understanding of RNA carriage by ejaculate spermatozoa. Human Reproduction Update 12 757–767. (10.1093/humupd/dml037) 16882702

[bib112] Miller D Ostermeier GC Krawetz SA 2005 The controversy, potential and roles of spermatozoal RNA. Trends in Molecular Medicine 11 156–163. (10.1016/j.molmed.2005.02.006) 15823753

[bib113] Moll AC Imhof SM Schouten-van Meeteren AY van Leeuwen FE 2003 In-vitro fertilisation and retinoblastoma. Lancet 361 1392. (10.1016/S0140-6736(03)13065-6) 12711501

[bib114] Montjean D De La Grange P Gentien D Rapinat A Belloc S Cohen-Bacrie P Menezo Y Benkhalifa M 2012 Sperm transcriptome profiling in oligozoospermia. Journal of Assisted Reproduction and Genetics 29 3–10. (10.1007/s10815-011-9644-3) 21989496 PMC3252406

[bib115] Moomjy M Colombero LT Veeck LL Rosenwaks Z Palermo GD 1999 Sperm integrity is critical for normal mitotic division and early embryonic development. Molecular Human Reproduction 5 836–844. (10.1093/molehr/5.9.836) 10460222

[bib116] Nagy ZP Liu J Joris H Verheyen G Tournaye H Camus M Derde MP Devroey P Van Steirteghem AC 1995 Andrology: the result of intracytoplasmic sperm injection is not related to any of the three basic sperm parameters. Human Reproduction 10 1123–1129. (10.1093/oxfordjournals.humrep.a136104) 7657751

[bib117] Nakasuji T Ogonuki N Chiba T Kato T Shiozawa K Yamatoya K Tanaka H Kondo T Miyado K Miyasaka N 2017 Complementary critical functions of Zfy1 and Zfy2 in mouse spermatogenesis and reproduction. PLoS Genetics 13 e1006578. (10.1371/journal.pgen.1006578) 28114340 PMC5287576

[bib118] Neri QV 2010 Tweaking Human Fertilization. New York, NY: Weill Cornell Medical College.

[bib119] Neri QV Monahan D Kocent J Hu JCY Rosenwaks Z Palermo GD 2010 Assessing and restoring sperm fertilizing competence. Fertility and Sterility 94 S147. (10.1016/j.fertnstert.2010.07.589)

[bib120] Neri QV Scala V Rosenwaks Z Palermo GD 2011 Assessment of the sperm centrosome. Fertility and Sterility 96 S235–S236. (10.1016/j.fertnstert.2011.07.904)

[bib121] Neri QV Hu J Rosenwaks Z Palermo GD 2014a Understanding the spermatozoon. Methods in Molecular Biology 1154 91–119. (10.1007/978-1-4939-0659-8_5) 24782007

[bib122] Neri QV Lee B Rosenwaks Z Machaca K Palermo GD 2014b Understanding fertilization through intracytoplasmic sperm injection (ICSI). Cell Calcium 55 24–37. (10.1016/j.ceca.2013.10.006) 24290744 PMC4046257

[bib123] Neuwinger J Behre HM Nieschlag E 1990 External quality control in the andrology laboratory: an experimental multicenter trial. Fertility and Sterility 54 308–314. (10.1016/S0015-0282(16)53709-1) 2379629

[bib124] Noblanc A Damon-Soubeyrand C Karrich B Henry-Berger J Cadet R Saez F Guiton R Janny L Pons-Rejraji H Alvarez JG 2013 DNA oxidative damage in mammalian spermatozoa: where and why is the male nucleus affected? Free Radical Biology and Medicine 65 719-723. (10.1016/j.freeradbiomed.2013.07.044) 23954469

[bib125] Nyboe Andersen A Goossens V Bhattacharya S Ferraretti AP Kupka MS de Mouzon J Nygren KG 2009 Assisted reproductive technology and intrauterine inseminations in Europe, 2005: results generated from European registers by ESHRE: ESHRE. The European IVF Monitoring Programme (EIM), for the European Society of Human Reproduction and Embryology (ESHRE). Human Reproduction 24 1267–1287. (10.1093/humrep/dep035) 19225009

[bib126] Ohlander S Hotaling J Kirshenbaum E Niederberger C Eisenberg ML 2014 Impact of fresh versus cryopreserved testicular sperm upon intracytoplasmic sperm injection pregnancy outcomes in men with azoospermia due to spermatogenic dysfunction: a meta-analysis. Fertility and Sterility 101 344–349. (10.1016/j.fertnstert.2013.10.012) 24345355

[bib127] Oliveira JBA Cavagna M Petersen CG Mauri AL Massaro FC Silva LFI Baruffi RLR Franco JG 2011 Pregnancy outcomes in women with repeated implantation failures after intracytoplasmic morphologically selected sperm injection (IMSI). Reproductive Biology and Endocrinology 9 99–99. (10.1186/1477-7827-9-99) 21781299 PMC3156729

[bib128] Orstavik KH 2003 Intracytoplasmic sperm injection and congenital syndromes because of imprinting defects. Tidsskrift for Den Norske Laegeforening 123 177. 12607501

[bib129] Ostermeier GC Dix DJ Miller D Khatri P Krawetz SA 2002 Spermatozoal RNA profiles of normal fertile men. Lancet 360 772–777. (10.1016/S0140-6736(02)09899-9) 12241836

[bib130] Ostermeier GC Goodrich RJ Diamond MP Dix DJ Krawetz SA 2005a Toward using stable spermatozoal RNAs for prognostic assessment of male factor fertility. Fertility and Sterility 83 1687–1694. (10.1016/j.fertnstert.2004.12.046) 15950637

[bib131] Ostermeier GC Goodrich RJ Moldenhauer JS Diamond MP Krawetz SA 2005b A suite of novel human spermatozoal RNAs. Journal of Andrology 26 70–74. 15611569

[bib132] Pabuccu EG Caglar GS Tangal S Haliloglu AH Pabuccu R 2017 Testicular versus ejaculated spermatozoa in ICSI cycles of normozoospermic men with high sperm DNA fragmentation and previous ART failures. Andrologia 49. (10.1111/and.12609) 27108915

[bib133] Palermo G Van Steirteghem A 1991 Enhancement of acrosome reaction and subzonal insemination of a single spermatozoon in mouse eggs. Molecular Reproduction and Development 30 339–345. (10.1002/mrd.1080300408) 1751038

[bib134] Palermo G Joris H Devroey P Van Steirteghem AC 1992a Induction of acrosome reaction in human spermatozoa used for subzonal insemination. Human Reproduction 7 248–254. (10.1093/oxfordjournals.humrep.a137626) 1577940

[bib135] Palermo G Joris H Devroey P Van Steirteghem AC 1992b Pregnancies after intracytoplasmic injection of single spermatozoon into an oocyte. Lancet 340 17–18. (10.1016/0140-6736(92)92425-F) 1351601

[bib136] Palermo G Munne S Cohen J 1994 The human zygote inherits its mitotic potential from the male gamete. Human Reproduction 9 1220–1225. (10.1093/oxfordjournals.humrep.a138682) 7962421

[bib137] Palermo GD Cohen J Alikani M Adler A Rosenwaks Z 1995 Intracytoplasmic sperm injection: a novel treatment for all forms of male factor infertility. Fertility and Sterility 63 9. (10.1016/s0015-0282(16)57603-1) 7750593

[bib138] Palermo GD Cohen J Rosenwaks Z 1996a Intracytoplasmic sperm injection: a powerful tool to overcome fertilization failure. Fertility and Sterility 65 899–908. (10.1016/S0015-0282(16)58257-0) 8612845

[bib139] Palermo GD Colombero LT Schattman GL Davis OK Rosenwaks Z 1996b Evolution of pregnancies and initial follow-up of newborns delivered after intracytoplasmic sperm injection. JAMA 276 1893–1897. (10.1001/jama.1996.03540230043033) 8968015

[bib140] Palermo GD Schlegel PN Colombero LT Zaninovic N Moy F Rosenwaks Z 1996c Aggressive sperm immobilization prior to intracytoplasmic sperm injection with immature spermatozoa improves fertilization and pregnancy rates. Human Reproduction 11 1023–1029. (10.1093/oxfordjournals.humrep.a019290) 8671384

[bib141] Palermo GD Avrech OM Colombero LT Wu H Wolny YM Fissore RA Rosenwaks Z 1997 Human sperm cytosolic factor triggers Ca2+ oscillations and overcomes activation failure of mammalian oocytes. Molecular Human Reproduction 3 367–374. (10.1093/molehr/3.4.367) 9237265

[bib142] Palermo GD Schlegel PN Hariprashad JJ Ergün B Mielnik A Zaninovic N Veeck LL Rosenwaks Z 1999 Fertilization and pregnancy outcome with intracytoplasmic sperm injection for azoospermic men. Human Reproduction 14 741–748. (10.1093/humrep/14.3.741) 10221707

[bib143] Palermo GD Neri QV Takeuchi T Rosenwaks Z 2009 ICSI: where we have been and where we are going. Seminars in Reproductive Medicine 27 191–201. (10.1055/s-0029-1202309) 19247922

[bib144] Palermo GD Hu JCY Rienzi L Maggiulli R Takeuchi T Yoshida A Tanaka A Kusunoki H Watanabe S Neri QV 2011 Thoughts on IMSI. In Biennial Review of Infertility. Ed CRacowsky. Boston, MA: Springer Science+Business Media, LLC. (10.1007/978-1-4419-8456-2_20)

[bib145] Palermo GD Neri QV Monahan D Kocent J Rosenwaks Z 2012 Development and current applications of assisted fertilization. Fertility and Sterility 97 248–259. (10.1016/j.fertnstert.2011.12.037) 22289284

[bib146] Palermo GD Neri QV Fields T Rosenwaks Z 2013 Popularity of ICSI. In Biennial Review of Infertility. Ed PNSchlegel. New York: Springer Sciences. (10.1007/978-1-4614-7187-5_19)

[bib147] Palermo GD Kocent J Monahan D Neri QV Rosenwaks Z 2014a Treatment of male infertility. Methods in Molecular Biology 1154 385–405. (10.1007/978-1-4939-0659-8_18) 24782020

[bib148] Palermo GD Kocent J Monahan D Neri QV Rosenwaks Z 2014b Treatment of male infertility. Methods in Molecular Biology 1154 20. (10.1007/978-1-4939-0659-8_18) 24782020

[bib149] Palermo GD Neri QV Cozzubbo T Rosenwaks Z 2014c Perspectives on the assessment of human sperm chromatin integrity. Fertility and Sterility 102 1508–1517. (10.1016/j.fertnstert.2014.10.008) 25456796

[bib150] Palermo GD Neri QV Schlegel PN Rosenwaks Z 2014d Intracytoplasmic sperm injection (ICSI) in extreme cases of male infertility. PLoS ONE 9 e113671. (10.1371/journal.pone.0113671) 25437298 PMC4249967

[bib151] Palermo GD Cheung S Cozzubbo T Neri QV Rosenwaks Z 2015a The ideal spermatozoon for ART. In Biennial Review of Infertility. Ed DTCarrell. Switzerland: Springer International. (10.1007/978-3-319-17849-3_9)

[bib152] Palermo GD Neri QV Rosenwaks Z 2015b To ICSI or Not to ICSI. Seminars in Reproductive Medicine 33 92–102. (10.1055/s-0035-1546825) 25734347

[bib153] Patrizio P Zielenski J 1996 Congenital absence of the vas deferens: a mild form of cystic fibrosis. Molecular Medicine Today 2 24–31. (10.1016/1357-4310(96)88755-7) 8796848

[bib154] Peña JE Klein J Thornton M II Chang PL Sauer MV 2002 Successive pregnancies with delivery of two healthy infants in a couple who was discordant for human immunodeficiency virus infection. Fertility and Sterility 78 421–423. (10.1016/s0015-0282(02)03213-2) 12137886

[bib155] Pereira N Cozzubbo T Cheung S Rosenwaks Z Palermo GD Neri QV 2016a Identifying maternal constraints on fetal growth and subsequent perinatal outcomes using a multiple embryo implantation model. PLoS ONE 11 e0166222. (10.1371/journal.pone.0166222) 27824942 PMC5100992

[bib156] Pereira N Neri QV Lekovich JP Palermo GD Rosenwaks Z 2016b The role of in-vivo and in-vitro maturation time on ooplasmic dysmaturity. Reproductive BioMedicine Online 32 401–406. (10.1016/j.rbmo.2016.01.007) 26896430

[bib157] Plastira K Msaouel P Angelopoulou R Zanioti K Plastiras A Pothos A Bolaris S Paparisteidis N Mantas D 2007 The effects of age on DNA fragmentation, chromatin packaging and conventional semen parameters in spermatozoa of oligoasthenoteratozoospermic patients. Journal of Assisted Reproduction and Genetics 24 437–443. (10.1007/s10815-007-9162-5) 17768675 PMC3455076

[bib158] Platts AE Dix DJ Chemes HE Thompson KE Goodrich R Rockett JC Rawe VY Quintana S Diamond MP Strader LF 2007 Success and failure in human spermatogenesis as revealed by teratozoospermic RNAs. Human Molecular Genetics 16 763–773. (10.1093/hmg/ddm012) 17327269

[bib159] Porcu E Fabbri R Seracchioli R Ciotti PM Magrini O Flamigni C 1997 Birth of a healthy female after intracytoplasmic sperm injection of cryopreserved human oocytes. Fertility and Sterility 68 724–726. (10.1016/S0015-0282(97)00268-9) 9341619

[bib160] Rivera RM Stein P Weaver JR Mager J Schultz RM Bartolomei MS 2008 Manipulations of mouse embryos prior to implantation result in aberrant expression of imprinted genes on day 9.5 of development. Human Molecular Genetics 17 1–14. (10.1093/hmg/ddm280) 17901045

[bib161] Ron-El R Strassburger D Friedler S Komarovski D Bern O Soffer Y Raziel A 1997 Extended sperm preparation: an alternative to testicular sperm extraction in non-obstructive azoospermia. Human Reproduction 12 1222–1226. (10.1093/humrep/12.6.1222) 9222005

[bib162] Rousseaux S Hazzouri M Pelletier R Monteil M Usson Y Sele B 1998 Disomy rates for chromosomes 14 and 21 studied by fluorescent in-situ hybridization in spermatozoa from three men over 60 years of age. Molecular Human Reproduction 4 695–699. (10.1093/molehr/4.7.695) 9701792

[bib163] Royo H Seitz H ElInati E Peters AHFM Stadler MB Turner JMA 2015 Silencing of X-linked microRNAs by meiotic sex chromosome inactivation. PLOS Genetics 11 e1005461. (10.1371/journal.pgen.1005461) 26509798 PMC4624941

[bib164] Sakkas D Alvarez JG 2010 Sperm DNA fragmentation: mechanisms of origin, impact on reproductive outcome, and analysis. Fertility and Sterility 93 1027–1036. (10.1016/j.fertnstert.2009.10.046) 20080235

[bib165] Saleh RA Agarwal A Sharma RK Said TM Sikka SC Thomas AJ Jr 2003 Evaluation of nuclear DNA damage in spermatozoa from infertile men with varicocele. Fertility and Sterility 80 1431–1436. (10.1016/S0015-0282(03)02211-8) 14667879

[bib166] Sathananthan AH Kola I Osborne J Trounson A Ng SC Bongso A Ratnam SS 1991 Centrioles in the beginning of human development. PNAS 88 4806–4810. (10.1073/pnas.88.11.4806) 2052559 PMC51755

[bib167] Sathananthan AH Ratnam SS Ng SC Tarin JJ Gianaroli L Trounson A 1996 The sperm centriole: its inheritance, replication and perpetuation in early human embryos. Human Reproduction 11 345–356. (10.1093/HUMREP/11.2.345) 8671223

[bib168] Sauer MV Chang PL 2002 Establishing a clinical program for human immunodeficiency virus 1–seropositive men to father seronegative children by means of in vitro fertilization with intracytoplasmic sperm injection. American Journal of Obstetrics and Gynecology 186 627–633. (10.1067/mob.2002.122125) 11967483

[bib169] Schachter-Safrai N Karavani G Levitas E Friger M Zeadna A Lunenfeld E Har-Vardi I 2017 Does cryopreservation of sperm affect fertilization in nonobstructive azoospermia or cryptozoospermia? Fertility and Sterility 107 1148–1152. (10.1016/j.fertnstert.2017.03.009) 28392002

[bib170] Schalkoff ME Oskowitz SP Powers RD 1989 Ultrastructural observations of human and mouse oocytes treated with cryopreservatives. Biology of Reproduction 40 14. (10.1095/biolreprod40.2.379) 2720033

[bib171] Schieve LA Meikle SF Ferre C Peterson HB Jeng G Wilcox LS 2002 Low and very low birth weight in infants conceived with use of assisted reproductive technology. New England Journal of Medicine 346 731–737. (10.1056/NEJMoa010806) 11882728

[bib172] Schieve LA Rasmussen SA Buck GM Schendel DE Reynolds MA Wright VC 2004 Are children born after assisted reproductive technology at increased risk for adverse health outcomes? Obstetrics and Gynecology 103 1154–1163. (10.1097/01.AOG.0000124571.04890.67) 15172847

[bib173] Schlegel PN 2009 Nonobstructive azoospermia: a revolutionary surgical approach and results. Seminars in Reproductive Medicine 27 165–170. (10.1055/s-0029-1202305) 19247918

[bib174] Schlegel PN Li PS 1998 Microdissection TESE: sperm retrieval in non-obstructive azoospermia. Human Reproduction Update 4 439. (10.1093/humupd/4.4.439) 9825858

[bib175] Schlegel PN Palermo GD Goldstein M Menendez S Zaninovic N Veeck LL Rosenwaks Z 1997 Testicular sperm extraction with intracytoplasmic sperm injection for nonobstructive azoospermia. Urology 49 435–440. (10.1016/S0090-4295(97)00032-0) 9123710

[bib176] Schuster A Tang C Xie Y Ortogero N Yuan S Yan W 2016 SpermBase: a database for sperm-borne RNA contents. Biology of Reproduction 95 99. (10.1095/biolreprod.116.142190) 27628216 PMC5178153

[bib177] Schwartz D Laplanche A Jouannet P David G 1979 Within-subject variability of human semen in regard to sperm count, volume, total number of spermatozoa and length of abstinence. Journal of Reproduction and Fertility 57 391–395. (10.1530/jrf.0.0570391) 513028

[bib178] Silber SJ Johnson L Verheyen G Van Steirteghem A 2000 Round spermatid injection. Fertility and Sterility 73 897–900. (10.1016/S0015-0282(00)00488-X) 10785213

[bib179] Simon L Lewis SE 2011 Sperm DNA damage or progressive motility: which one is the better predictor of fertilization in vitro? Systems Biology in Reproductive Medicine 57 133–138. (10.3109/19396368.2011.553984) 21299480

[bib180] Simon L Zini A Dyachenko A Ciampi A Carrell DT 2017 A systematic review and meta-analysis to determine the effect of sperm DNA damage on in vitro fertilization and intracytoplasmic sperm injection outcome. Asian Journal of Andrology 19 80–90. (10.4103/1008-682x.182822) 27345006 PMC5227680

[bib181] Sone Y Ito M Shirakawa H Shikano T Takeuchi H Kinoshita K Miyazaki S 2005 Nuclear translocation of phospholipase C-zeta, an egg-activating factor, during early embryonic development. Biochemical and Biophysical Research Communications 330 690–694. (10.1016/j.bbrc.2005.03.032) 15809052

[bib182] Sotolongo B Lino E Ward WS 2003 Ability of hamster spermatozoa to digest their own DNA1. Biology of Reproduction 69 2029–2035. (10.1095/biolreprod.103.020594) 12930713

[bib183] Sousa M Tesarik J 1994 Fertilization and early embryology: Ultrastructural analysis of fertilization failure after intracytoplasmic sperm injection. Human Reproduction 9 2374–2380. (10.1093/oxfordjournals.humrep.a138455) 7714161

[bib184] Stalf T Sánchez R Köhn FM Schalles U Kleinstein J Hinz V Tielsch J Khanaga O Turley H Gips H 1995 Pregnancy and birth after intracytoplasmic sperm injection with spermatozoa from a patient with tail stump syndrome. Human Reproduction 10 2112–2114. (10.1093/oxfordjournals.humrep.a136244) 8567850

[bib185] Steiner B Masood R Rufibach K Niedrist D Kundert O Riegel M Schinzel A 2015 An unexpected finding: younger fathers have a higher risk for offspring with chromosomal aneuploidies. European Journal of Human Genetics 23 466–472. (10.1038/ejhg.2014.122) 25005732 PMC4666566

[bib186] Sugaya S 2010 Pregnancy following calcium ionophore oocyte activation in an oligozoospermia patient with repeated failure of fertilization after ICSI. Clinical and Experimental Obstetrics and Gynecology 37 261–262. 21355452

[bib187] Sullivan EA Zegers-Hochschild F Mansour R Ishihara O de Mouzon J Nygren KG Adamson GD 2013 International Committee for Monitoring Assisted Reproductive Technologies (ICMART) world report: assisted reproductive technology 2004. Human Reproduction 28 1375–1390. (10.1093/humrep/debib36) 23442757

[bib188] Sun F Ko E Martin RH 2006 Is there a relationship between sperm chromosome abnormalities and sperm morphology? Reproductive Biology and Endocrinology 4 1. (10.1186/1477-7827-4-1) 16436209 PMC1395314

[bib189] Sunderam S Kissin DM Crawford SB Folger SG Jamieson DJ Warner L Barfield WD 2015 Assisted reproductive technology surveillance – United States. MMWR Surveillance Summaries 28. (10.15585/mmwr.ss6411a1) PMC5829717 28182605

[bib190] Sutcliffe AG Peters CJ Bowdin S Temple K Reardon W Wilson L Clayton-Smith J Brueton LA Bannister W Maher ER 2006 Assisted reproductive therapies and imprinting disorders--a preliminary British survey. Human Reproduction 21 1009–1011. (10.1093/humrep/dei405) 16361294

[bib191] Swann K Larman MG Saunders CM Lai FA 2004 The cytosolic sperm factor that triggers Ca2+ oscillations and egg activation in mammals is a novel phospholipase C: PLCzeta. Reproduction 127 431–439. (10.1530/rep.1.00169) 15047934

[bib192] Tanaka A Nagayoshi M Tanaka I Kusunoki H 2012 Human sperm head vacuoles are physiological structures formed during the sperm development and maturation process. Fertility and Sterility 98 315–320. (10.1016/j.fertnstert.2012.04.034) 22624673

[bib193] Tanaka A Nagayoshi M Takemoto Y Tanaka I Kusunoki H Watanabe S Kuroda K Takeda S Ito M Yanagimachi R 2015 Fourteen babies born after round spermatid injection into human oocytes. PNAS 112 14629–14634. (10.1073/pnas.1517466112) 26575628 PMC4664346

[bib194] Tang SS Gao H Zhao Y Ma S 2010 Aneuploidy and DNA fragmentation in morphologically abnormal sperm. International Journal of Andrology 33 e163–e179. (10.1111/j.1365-2605.2009.00982.x) 19732195

[bib195] Tosti E Ménézo Y 2016 Gamete activation: basic knowledge and clinical applications. Human Reproduction Update 22 420–439. (10.1093/humupd/dmw014) 27278231 PMC4917743

[bib196] Toyama Y Iwamoto T Yajima M Baba K Yuasa S 2000 Decapitated and decaudated spermatozoa in man, and pathogenesis based on the ultrastructure. International Journal of Andrology 23 109–115. (10.1046/j.1365-2605.2000.bib1-1-00217.x) 10762437

[bib197] Van Blerkom J Davis PW 1994 Cytogenetic, cellular, and developmental consequences of cryopreservation of immature and mature mouse and human oocytes. Microscopy Research and Technique 27 28. (10.1002/jemt.1070270209) 8123908

[bib198] Vanderzwalmen P Zech H Birkenfeld A Yemini M Bertin G Lejeune B Nijs M Segal L Stecher A Vandamme B van Roosendaal E Schoysman R 1997 Intracytoplasmic injection of spermatids retrieved from testicular tissue: influence of testicular pathology, type of selected spermatids and oocyte activation. Human Reproduction 12 1203–1213. (10.1093/humrep/12.6.1203) 9222002

[bib199] Vincent C Pickering SJ Johnson MH 1990 The hardening effect of dimethylsulphoxide on the mouse zona pellucida requires the presence of an oocyte and is associated with a reduction in the number of cortical granules present. Journal of Reproduction and Fertility 89 6. (10.1530/jrf.0.0890253) 2374118

[bib200] Vitorino RL Grinsztejn BG de Andrade CAF Hökerberg YHM de Souza CTV Friedman RK Passos SRL 2011 Systematic review of the effectiveness and safety of assisted reproduction techniques in couples serodiscordant for human immunodeficiency virus where the man is positive. Fertility and Sterility 95 1684–1690. (10.1016/j.fertnstert.2011.01.127) 21324449

[bib201] Vloeberghs V Verheyen G Haentjens P Goossens A Polyzos NP Tournaye H 2015 How successful is TESE-ICSI in couples with non-obstructive azoospermia? Human Reproduction 30 1790–1796. (10.1093/humrep/dev139) 26082482

[bib202] Wallach EE Palermo GD Cohen J Rosenwaks Z 1996 Intracytoplasmic sperm injection: a powerful tool to overcome fertilization failure. Fertility and Sterility 65 899–908. (10.1016/S0015-0282(16)58257-0) 8612845

[bib203] Ward WS 2010 Function of sperm chromatin structural elements in fertilization and development. MHR: Basic Science of Reproductive Medicine 16 30–36. (10.1093/molehr/gap080) 19748904 PMC2790366

[bib204] Watanabe S Tanaka A Fujii S Mizunuma H Fukui A Fukuhara R Nakamura R Yamada K Tanaka I Awata S 2011 An investigation of the potential effect of vacuoles in human sperm on DNA damage using a chromosome assay and the TUNEL assay. Human Reproduction 26 978–986. (10.1093/humrep/der047) 21362682

[bib205] WHO 2010 WHO Laboratory Manual for the Examination and Processing of Human Semen, 5th ed. Switzerland.

[bib206] Wilson TJ Lacham-Kaplan O Gould J Holloway A Bertoncello I Hertzog PJ Trounson A 2007 Comparison of mice born after intracytoplasmic sperm injection with in vitro fertilization and natural mating. Molecular Reproduction and Development 74 512–519. (10.1002/mrd.20644) 16998805

[bib207] Wolny YM Fissore RA Wu H Reis MM Colombero LT Ergun B Rosenwaks Z Palermo GD 1999 Human glucosamine-6-phosphate isomerase, a homologue of hamster oscillin, does not appear to be involved in Ca2+ release in mammalian oocytes. Molecular Reproduction and Development 52 277–287. (10.1002/(SICI)1098-2795(199903)52:3<277::AID-MRD5>3.0.CO;2-0) 10206659

[bib208] Wykes SM Krawetz SA 2003 The structural organization of sperm chromatin. Journal of Biological Chemistry 278 29471–29477. (10.1074/jbc.M304545200) 12775710

[bib209] Xing W Krishnamurthy H Sairam MR 2003 Role of follitropin receptor signaling in nuclear protein transitions and chromatin condensation during spermatogenesis. Biochemical and Biophysical Research Communications 312 697–701. (10.1016/j.bbrc.2003.10.177) 14680821

[bib210] Yanagida K 2004 Complete fertilization failure in ICSI. Human Cell 17 187–193. (10.1111/j.1749-0774.2004.tb00042.x) 16035503

[bib211] Younglai EV Holt D Brown P Jurisicova A Casper RF 2001 Sperm swim-up techniques and DNA fragmentation. Human Reproduction 16 1950–1953. (10.1093/humrep/16.9.1950) 11527903

[bib212] Zanko A Cozzubbo T Neri QV Rosenwaks Z Palermo GD 2014 Revisiting DNA integrity in function of sperm motility. Human Reproduction 29 1. (10.1093/humrep/det385) 24218402

[bib213] Zhang Q Li G Zhang L Sun X Zhang D Lu J Ma J Yan J Chen ZJ 2017 Maternal common variant rs2305957 spanning PLK4 is associated with blastocyst formation and early recurrent miscarriage. Fertility and Sterility 107 1034.e1035–1040.e1035. (10.1016/j.fertnstert.2017.01.006) 28238495

[bib214] Zini A 2011 Are sperm chromatin and DNA defects relevant in the clinic? Systems Biology in Reproductive Medicine 57 78–85. (10.3109/19396368.2010.515704) 21208147

[bib215] Zini A Sigman M 2009 Are tests of sperm DNA damage clinically useful? Pros and Cons. Journal of Andrology 30 219–229. (10.2164/jandrol.108.006908) 19059901

[bib216] Zini A Bielecki R Phang D Zenzes MT 2001 Correlations between two markers of sperm DNA integrity, DNA denaturation and DNA fragmentation, in fertile and infertile men. Fertility and Sterility 75 674–677. (10.1016/S0015-0282(00)01796-9) 11287017

[bib217] Zini A Boman JM Belzile E Ciampi A 2008 Sperm DNA damage is associated with an increased risk of pregnancy loss after IVF and ICSI: systematic review and meta-analysis. Human Reproduction 23 2663–2668. (10.1093/humrep/den321) 18757447

